# Exploring the impact of the environment on physical activity in patients with chronic obstructive pulmonary disease (EPCOT)—A comparative analysis between suggested and free walking: Protocol study

**DOI:** 10.1371/journal.pone.0306045

**Published:** 2024-08-13

**Authors:** Larissa Guimarães Paiva, Túlio Medina Dutra de Oliveira, Nara Batista de Souza, Klaus Chaves Alberto, Daniela Pereira Almeida, Cristino Carneiro Oliveira, Anderson José, Carla Malaguti

**Affiliations:** 1 Graduate Program of Rehabilitation Sciences and Physical and Functional Performance, Federal University of Juiz de Fora (UFJF) - Juiz de Fora, Juiz de Fora, Minas Gerais, Brazil; 2 Graduate Program in Health, Federal University of Juiz de Fora (UFJF) - Juiz de Fora, Juiz de Fora, Minas Gerais, Brazil; 3 Graduate Program in Built Environment, Federal University of Juiz de Fora (UFJF) - Juiz de Fora, Juiz de Fora, Minas Gerais, Brazil; 4 Graduate Program in Architecture and Urbanism, Federal University of Viçosa (UFV) - Viçosa, Viçosa, Minas Gerais, Brazil; 5 Graduate Program of Rehabilitation Sciences and Physical and Functional Performance, Federal University of Juiz de Fora (UFJF) - Governador Valadares, Governador Valadares, Minas Gerais, Brazil; 6 Graduation Program on Rehabilitation Sciences, Federal University of Minas Gerais, Belo Horizonte, Brazil; 7 Graduate Program of Rehabilitation Sciences and Physical and Functional Performance, Graduate Program in Health, Federal University of Juiz de Fora (UFJF) - Juiz de Fora, Juiz de Fora, Minas Gerais, Brazil; PLOS: Public Library of Science, UNITED KINGDOM

## Abstract

**Background:**

Individuals with chronic obstructive pulmonary disease (COPD) exhibit reduced levels of physical activity, which are associated with poorer outcomes. The number of clinical trials aiming to promote behavioral changes to increase physical activity in this population has grown; therefore, these trials have yet to produce satisfactory results. An ecological model encompassing individual, social, environmental, and political factors represent a potentially more effective approach to promoting physical activity. While favorable urban environments can positively impact physical activity, specifically tailored environmental interventions for individuals with COPD could enhance their engagement in physical activity. Therefore, the aim of this randomized controlled trial (RCT) study was to analyze the effects of walking in a suggested environment and free walking on physical activity levels in individuals with COPD.

**Methods:**

The environment on physical activity for chronic obstructive disease (EPCOT) is a randomized controlled clinical trial protocol approved by our institution’s Ethics Committee and registered with The Brazilian Registry of Clinical Trials (ReBEC) (https://ensaiosclinicos.gov.br, number RBR-4tfwdhp). This protocol will involve 38 volunteers diagnosed with COPD recruited from the pulmonary physiotherapy and rehabilitation service. The volunteers were randomly divided into two walking groups: an experimental group (ERG) with guidance for walking in a suggested environment and an active control group (ACG) instructed to choose their own routes. The intervention consisted of eight consecutive weeks, with progressive walks carried out 3 to 5 times weekly. The primary outcome will be assessing participants’ physical activity levels. Secondary outcomes will include exercise capacity, quality of life, dyspnea levels, motivation, anxiety, depression, and perceptions of the environment. All assessments will occur before and after the intervention period, aiming to fill a literature gap by investigating the impact of urban environments on COPD-related physical activity. The results may shed light on the importance of environmental factors in promoting physical activity among individuals with COPD, helping to develop more effective interventions.

## Introduction

Physical inactivity is a common feature of many chronic diseases, both as a cause and a consequence. The high prevalence of physical inactivity is a problem [1, 2] that contributes to increased morbidity, premature death [3], and healthcare costs [4]. The World Health Organization (WHO) [5], the American College of Sports Medicine [6], and the Physical Activity Guidelines for the Brazilian Population [7] recommend accumulating 150 minutes of moderate physical activity per week or 75 minutes of vigorous physical activity, which translates into health-related benefits. Additionally, accumulating 7,500 to 10,000 steps per day is recommended for adults [8], and accumulating approximately 5,000 steps per day is recommended for older and special populations, such as those with chronic lung diseases [9].

Patients with chronic obstructive pulmonary disease (COPD) perform less physical activity than healthy controls [10, 11]. Existing data show that the amount of time spent walking is lower in patients with COPD than in healthy individuals of the same age [12–14]. Associations between physical activity and the clinical characteristics of COPD patients, such as disease severity, comorbidities, exacerbations, and behavioral factors, have also been observed [15]. Prospective longitudinal studies have shown an association between low levels of physical activity and an increased number of exacerbations, risk of hospitalization, and all-cause mortality in COPD patients [16, 17].

Studies have focused on testing the effectiveness of interventions such as pulmonary rehabilitation programs, pharmacological treatment, oxygen therapy, and behavioral interventions for increasing physical activity in COPD patients [18, 19]. However, these interventions have primarily been designed to target individual factors such as limiting symptoms, physical capacity, and overall health status, which have not resulted in lasting lifestyle changes for this population [19]. On the other hand, physical activity depends on various factors, including biological, behavioral, and genetic factors, as well as social, environmental, cultural, and public policy factors.

The ecological model of physical activity adopts a comprehensive perspective to understand inactive behavior, recognizing the influence of social and environmental factors, including external health components such as urban planning, transportation systems, squares, parks, and bike paths [20]. The urban environment plays a significant role in promoting physical activity, as living in cities that support exercise can help residents achieve approximately 45–59% of the recommended weekly guidelines for 150 minutes of physical activity [21].

Studies [22, 23] highlight that social, cultural, historical, and economic variations lead to distinct behaviors in population groups, reflected in people’s lifestyles influenced by family experiences, educational settings, and specific regional contexts. Therefore, adherence to specific behavioral patterns results from complex interactions between individual and contextual factors. "Walkability" is a concept that assesses the suitability of routes for walking and active transportation [24]. This subject has garnered growing attention in health research, spanning diverse populations ranging from older [25] to children to adolescents [26] and addressing various health conditions, such as diabetes risk [27], obesity [27, 28], and cardiovascular risk [29–31]. These investigations offer valuable insights into the influence of urban infrastructure on physical behavior and overall health. Walkability employs a combination of quantitative and qualitative methods to improve the allure and safety of streets and neighborhoods, ultimately fostering pedestrian mobility.

Three studies [32–34] addressed different strategies to promote physical activity in COPD patients. The first investigated the effectiveness of "Urban Training", revealing an increase in physical activity after 12 months [32]. The second study implemented urban walking circuits following rehabilitation in COPD patients, resulting in an average increase of 32.4 minutes of physical activity per day in the urban circuit group compared to the control group [33]. The third study focused on low-income and ethnically diverse adults in the U.S. introduced the "Peer Empowerment Program 4 Physical Activity" to promote physical activity among adults older than 50 years, highlighting the potential of peer-led approaches in community programs [34]. These trials highlight the importance of using predefined and contextualized routes to promote physical activity. However, urban circuits were not personalized in these studies according to the best walkability and participant preference.

Based on these premises, we hypothesize that a suggested route of better walkability, tailored and selected according to individual preferences, could encourage individuals with COPD to increase and maintain walking physical activity compared to a control group with free walking. Therefore, the aim of this randomized controlled trial (RCT) study was to analyze the effects of walking in a suggested environment and free walking on physical activity levels in individuals with COPD.

### Study design

The environment on physical activity for chronic obstructive disease (EPCOT) is a protocol for a parallel, two-group, randomized controlled trial that used concealed allocation with a 1:1 ratio and intention-to-treat analysis designed by independent investigators. The trial was registered at The Brazilian Registry of Clinical Trials (ReBEC) (https://ensaiosclinicos.gov.br, number RBR-4tfwdhp update on 04/12/2024) and follows the guidelines of the *Standard Protocol Items*: *Recommendations for Interventional Trials (SPIRIT)* [35, 36] (S1 Appendix).

The study protocol conformed to the ethical guidelines of the Declaration of Helsinki and was approved by the Research Ethics Committee of University Hospital of the Federal University of Juiz de Fora/MG (Approval Number: #5.889.099, on March 29, 2023). Written informed consent will be obtained from all participants.

Eligible participants received detailed information about the study’s objectives, risks, and benefits from the assessors. After reading and signing the informed consent form (S2 and S3 Appendices), participants provided consent to participate in the study, as established by Resolution 580/2018 of the National Health Council of Brazil. To ensure the privacy and confidentiality of the collected data, appropriate measures will be taken by the entire research team. The principal investigator safeguarded the information’s security by using codes instead of participants’ identifying data and controlling access to electronic files through a unique password. The data collected and analyzed by this Project will be disseminated at congresses and through international peer-reviewed journals and will not be reported in any of the forms of dissemination in this study.

**Fig 1:**
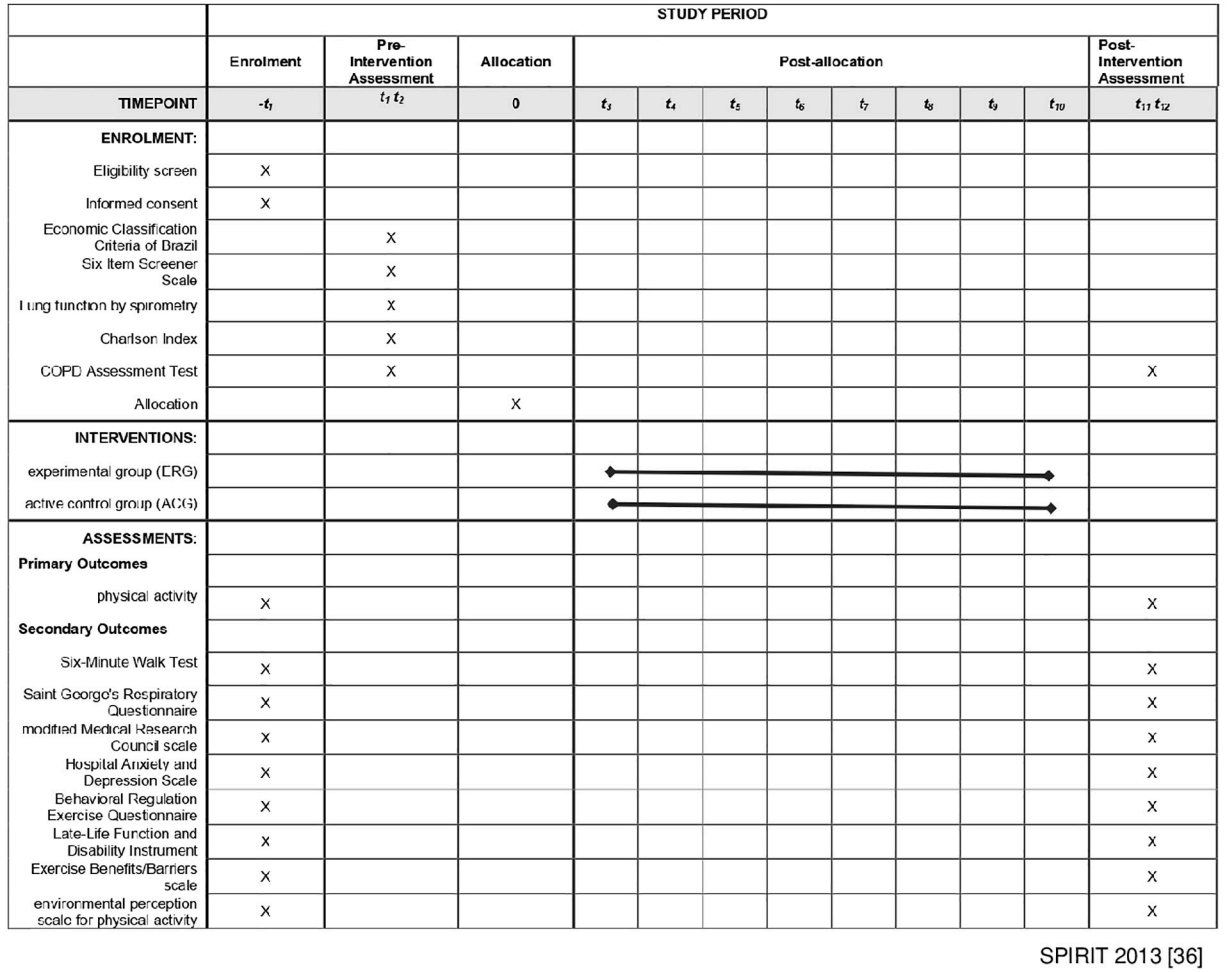
SPIRIT diagram. The figure details the timing of enrollment activities, intervention allocation, and assessments of outcomes over the course of the clinical trial. SPIRIT, Standard Protocol Items: Recommendations for Interventional Trials.

Random sampling will form two groups: an experimental group (ERG), which will engage in physical activity through walking on suggested routes with better walkability, and an active control group (ACG), which will be instructed to choose their own routes. Previously trained researchers will carry out the study. Primary and secondary outcome measures will be assessed two days, 48 hours apart, to prevent participant exhaustion. After an 8-week intervention period, reassessment will be performed (Fig 1).

### Sample size calculation

The sample size was calculated based on the primary outcome (physical activity) to detect a difference of at least 30 minutes in the weekly average physical activity between groups [33]. The analysis was conducted using G*Power 3.0.10 software (University of Kiel, Kiel, Germany) and considered the following parameters: a two-tailed test, a significance level (alpha) of 5%, and a statistical power of 90%. Based on these criteria, a minimum sample of 36 individuals was required for the study, with 16 participants per group, assuming an effect size of 1.03. To accommodate potential participant attrition and ensure the robustness of the results, an additional 20% was added to the calculated sample size. Therefore, the study will be conducted with a total sample of 44 participants.

### Participants

We will recruit individuals who meet the study’s eligibility criteria from the waiting list for the physiotherapy and pulmonary rehabilitation service at the University Hospital of the Federal University of Juiz de Fora through telephone or in-person contact. Additionally, individuals from primary healthcare units in the municipality of Juiz de Fora will be invited to participate. Recruitment will be carried out using leaflets distributed at the hospital, university, and on social media. These materials will guide potential participants who consider themselves eligible to contact a researcher involved in the study. Subsequently, a researcher blinded, unaware of the group assignment, will gather initial data to confirm eligibility. All data collection steps will be carried out in the Cardiorrespiratory Laboratory, located at the Faculty of Physiotherapy at Federal University of Juiz de Fora.

### Eligibility criteria

Individuals eligible for inclusion in this study were adults and elderly and had a confirmed diagnosis of COPD based on the criteria outlined by the Global Initiative for Chronic Obstructive Lung Disease (GOLD) [37]. Specifically, patients exhibit a postbronchodilator forced expiratory volume in one second (FEV_1_)/forced vital capacity (FVC) ratio <70% and an FEV_1_ <80% of the predicted value [37]. These individuals must be clinically stable [38] and free from unstable cardiovascular diseases, neurological disorders, or musculoskeletal conditions that might impede the proposed assessments and interventions. The exclusion criteria included individuals unable to perform or comprehend the study assessments due to physical or psychological impairments, those with a primary diagnosis of a respiratory disease other than COPD, individuals requiring oxygen therapy, those with a recent hospitalization within the last 3 months, participants in a physical activity program in the last 6 months, and those with identified cognitive impairment scoring equal to or less than 4 on the Six Item Screener [39].

### Randomization and allocation

Randomization will be performed with a 1:1 allocation ratio for the experimental or control group using random allocation software. The allocation sequence will be kept in identical, opaque, sealed, sequentially numbered envelopes and will remain concealed until the end of the evaluation. The responsible researcher will be instructed not to reveal the group assignments to the physiotherapist or other researchers involved in the study until its completion.

### Blinding

A "double-blind" approach will be adopted, where study participants and researchers responsible for data collection and analysis will be kept blind to the intervention received by each participant. To ensure the integrity of the blinding, an independent committee consisting of trained physiotherapists will be established for each stage of the research. Evaluating and reassessing researchers will not be aware of the intervention assigned to each participant. Instead, another professional will be designated to provide guidance and monitor the intervention, ensuring that the researchers do not have knowledge of the interventions received by the participants.

To ensure participant blinding, both groups will be blinded to the specific intervention. Participants will not be informed about the difference in route guidance, and furthermore, the walkers responsible for guiding the group will be instructed not to discuss or disclose any details of the intervention. This approach creates an environment in which participants have no knowledge or clues revealing the specific nature of the intervention, thus ensuring effective blinding of the study.

For statistical analysis and result interpretation, a researcher will be designated and will be blinded to the intervention assigned to each participant. Patients will not have access to information that could identify treatment groups. This ensures that the researcher cannot influence the results based on expectations or prior knowledge, promoting impartiality in data analysis.

### Intervention

Participants allocated to the experimental group will receive personalized and individual routes of better walkability, developed by an architect and urban planner through a virtual urban environmental analysis using the Microscale Audit of Pedestrian Streets (MAPS) [40], to collect audit data on the pedestrian environment and walkability in neighborhoods. The analysis will be performed through a geographical map based on satellite positioning from the participant’s residence. The architect and urban planner will conduct an interview with the participant to detect information that is not identified through virtual urban environmental analysis and will present the suggested routes combined with the participant’s preferences, choices, and acceptance. Participants in this group will receive 5 personalized circuits, with durations of approximately 15 minutes, 20 minutes, 30 minutes, 40 minutes, and 50 minutes, along with distances in kilometers. Each route includes a marked map of the path, a description of the start and end, and information about nearby cultural attractions or commercial areas. On the other hand, the control group will receive only guidance for walking without a specific route suggestion, allowing each participant to freely choose their walking path. The intervention period will last for 8 weeks, and participants will be advised to walk 3 to 5 times a week. The progression will follow the following schedule: in the first week, 15 minutes/day; in the second week, 20 minutes/day; in the third week, 30 minutes/day; in the fourth week, 40 minutes/day; and in the fifth week, 50 minutes/day until the eighth week.

To encourage walking, each participant in both groups received a special kit to assist them in their physical activity and well-being. The kit included a Yamax Pw 610 Pedometer, along with instructions on how to use it correctly. This device measures the number of steps taken during a walking session. Participants will also receive a step diary to record their walking time, the number of steps observed on the pedometer, and their perception of dyspnea and fatigue during the walk using the modified Borg Rating of Perceived Exertion scale [41]. This initiative allows participants to track and monitor their daily progress, motivating them to achieve personal goals and establish healthy habits. Additionally, a booklet containing recommendations such as the benefits of walking, energy conservation techniques, and stretches to be followed will be provided, aiming to optimize the walking experience (S4 Appendix).

Furthermore, two virtual health education sessions will be offered, which will be conducted in the first and third weeks of the intervention through videos or audio; the sessions will be sent to the participants or in a format that best suits their adherence. These educational sessions aimed to deepen participants’ knowledge about COPD, clinical control, symptom management, the importance of correct medication use, and the benefits of walking. This approach aims to empower participants with valuable information, useful tools, and ongoing support so that they can incorporate walking as part of a healthy lifestyle.

Throughout the program, both groups will receive indirect supervision through biweekly phone calls or text messages, where a professional will be available to answer questions, motivate, and monitor the progress of each individual.

### Sample characterization

To obtain a more precise characterization of the patient’s socioeconomic situation, the questionnaire of the Economic Classification Criteria of Brazil [42] was used. This instrument consists of a series of questions and is widely used in studies involving the Brazilian population. The questionnaire assesses the socioeconomic level of families, considering items such as purchasing power, possession of durable goods, and the level of education of the head of the family. Additionally, the Six Item Screener Scale [39] will be used to screen for cognitive impairment, involving words and questions related to temporal orientation.

Lung function will be measured by spirometry to confirm obstructive ventilation disorder in patients with COPD using a portable spirometer (Spirobank II, Medical International Research, New Berlin, USA) to obtain variables such as forced vital capacity (FVC), forced expiratory volume in the first second (FEV_1_), and the FEV_1_/FVC ratio, expressed in both absolute values and as a percentage of the predicted value [43]. Participants will be instructed to perform three maximum forced expiratory maneuvers, maintaining expiration during the expiratory flow period. We will strictly adhere to the acceptance and reproducibility criteria proposed by the guidelines [44] to ensure the quality of the examinations.

The Charlson comorbidity index [45], a tool that covers 19 different clinical conditions, was used to assess the presence of comorbidities. Each condition was categorized and scored based on its prognostic impact and associated mortality. For each group of clinical conditions, a relative risk was assigned, and these risks were converted into scores ranging from 0 to 6. Additionally, each patient’s age range is associated with a score ranging from 1 to 4. Thus, the result is obtained by summing the scores assigned to each clinical condition and the patient’s age range. A higher final score indicates a greater impact of comorbidities and a greater risk of complications on the patient’s prognosis.

The impact of COPD symptoms will be assessed using the COPD Assessment Test (CAT) [46], an instrument that quantifies how COPD symptoms affect the daily lives of patients. The CAT comprises eight items addressing various aspects of symptoms, including cough, sputum production, chest tightness, shortness of breath, limitations in daily activities, confidence in leaving the house, sleep quality, and energy level. CAT results can vary and are classified in terms of the clinical impact of symptoms as follows: 6–10 points (mild), 11–20 points (moderate), 21–30 points (severe), and 31–40 points (very severe) [46].

### Primary outcomes

An objective evaluation of participants’ daily physical activity levels will be conducted using the Actigraph GT3X^®^ accelerometer (Actigraph LLC, USA). This physical activity monitoring tool has been validated and found to be reliable for use in individuals with COPD [47]. The level of physical activity will be assessed by measuring the amount of time spent in minutes in light, moderate, and vigorous activity categories, as well as the total time, in addition to the daily step count and indicators of energy expenditure [10]. The collected data will be processed, recorded, and stored in the device’s internal memory, generating a report that will be analyzed on a computer using the specific ActiLife 6 software (Actigraph LLC, Pensacola, FL, USA) [48].

Before commencing accelerometer use, participants received instructions on how the device functions and its purpose. An elastic strap was worn to secure the accelerometer at the line of the iliac crest of the dominant lower limb. The accelerometer was worn continuously for a period of 7 consecutive days, with removal occurring only during bathing, water-related activities, and sleep. The device’s small size and minimal dimensions ensure comfort during use. Each participant will receive the accelerometer along with a manual containing information and instructions on the proper use of the device, as well as a diary recording the day of the week and the times the device is attached and removed. After the usage period, evaluators collected the device for data analysis.

The stored data will be analyzed using ActiLife 6 software (Actigraph LLC, Pensacola, FL, USA). The data will be collected at a frequency of 30 Hz and analyzed in 60-second epochs [49]. A day of data was considered valid if there was a minimum of 10 hours of use during 24 hours (from 0:00 to 23:59) [48]. The data from the first and last days were discarded, with a minimum of four days included in the analysis; at least one weekend day was included [50, 51].

The data analysis will be limited to waking hours, excluding any records during sleep. To calculate the minutes spent on moderate and vigorous activities per week, the data from all valid days were summed, adjusted for the total number of valid days, and multiplied to obtain the individual weekly average.

The step count will also be recorded, and patients can be classified into three categories based on step count: sedentary (<5,000 steps/day), not very active (5,000–7,499 steps/day), and active (≥7,500 steps/day) [52].

### Secondary outcomes

The secondary outcomes included exercise capacity, quality of life, dyspnea, anxiety and depression, exercise motivation, social participation, perceived benefits and barriers to physical activity, and environmental perception.

Exercise capacity will be assessed using the six-minute walk test (6MWT), following international guidelines for test administration [53], in a 30-meter-long corridor with a smooth surface. Participants will be instructed to walk as far as possible, as quickly as possible, without running for six minutes. Every minute, the examiner will inform the participants of the time remaining and provide standardized words of encouragement. Participants may rest if needed, but the timer will not be stopped. Two tests will be conducted with a 30-minute rest interval between them [54]. The test with the longest distance covered was used for analysis. Heart rate and pulse hemoglobin saturation (SpO2) will be continuously monitored using a portable pulse oximeter (MD Rossmax UT-100). Systemic blood pressure, dyspnea symptoms, and fatigue will also be measured before and after the test using the modified Borg Rating of Perceived Exertion scale [41]. The test may be stopped by the participant or examiner if the patient has discomfort, nausea, significant dyspnea, extreme fatigue, chest pain, or headache or if her SpO2 is <85%. At the end of the test, the distance covered was recorded in meters, and the percentage of the predicted distance reached was calculated [55]. An improvement in exercise capacity is expected, as indicated by an increase of at least 30 meters in the intervention group compared to the control group [53]. These data will be collected one week before the intervention’s start and after its completion.

Quality of life will be assessed using the Saint George’s Respiratory Questionnaire (SGRQ), which is specific for respiratory diseases and has been translated, culturally adapted, and validated for the Brazilian population [56]. The instrument addresses aspects related to three domains: symptoms, activity, and psychosocial impacts of the respiratory disease on the patient. Each domain has a maximum possible score, and the total score and domain scores range from 0 to 100. A lower score indicates a better quality of life [55]. In the context of the intervention, a significant improvement in the quality of life of the intervention group compared to the control group was expected, which may be evidenced by a 4-point reduction in the total SGRQ score [57].

Participants will report their dyspnea according to the modified Medical Research Council scale (mMRC), which has been translated, culturally adapted, and validated for the Brazilian population. This scale consists of five scores, with dyspnea grading ranging from 0 to 4, where a higher score indicates more pronounced symptoms of dyspnea [58].

Anxiety and depression will be measured using the Hospital Anxiety and Depression Scale (HADS). This scale was developed to estimate the prevalence of anxiety and depression in adults and is currently used in patients with chronic lung diseases [59]. The scale aims to identify cases (possible or probable) of anxiety and/or depression. It consists of 14 items divided into two subscales: seven questions for diagnosing anxiety disorders and another seven for diagnosing depressive disorders. Each question is scored from zero to three points (from absent to very frequent), with a maximum score of 21 points per subscale. Higher scores indicate greater severity of anxiety and depression [59]. A noticeable improvement is expected, represented by a reduction of 1.7 points in the anxiety subscale and 1.5 points in the depression subscale in the intervention group compared to the control group [60]. A clear improvement is expected, as evidenced by a reduction of 2 points in each subscale of anxiety and depression; these data were collected one week before and one week after the intervention.

The Behavioral Regulation Exercise Questionnaire (BREQ) will be used to assess patterns of self-determined behavior in physical exercise practice. It was created in 1997 [61] and was subsequently adapted in two ways. The most recent version is the BREQ-3 [62, 63], translated and adapted for the Portuguese language [63]. It consists of 23 items preceded by the following statement: "Why do you engage in physical exercise?" The respondents answered on a 5-point Likert scale indicating the degree to which they agreed that best fit their situation, ranging from "not true for me" (0) to "very true for me" (4). These 23 items are organized into six domains: motivation, external regulation, introjected regulation, identified regulation, integrated regulation (extrinsic motivation), and intrinsic regulation (intrinsic motivation). The six subscales that make up the BREQ-3 allow for the analysis of the motivation profile for physical exercise practice through the Self-Determination Index (SDI), where different weights are assigned to each subscale, with autonomous subscales receiving positive weights and less self-determined subscales receiving negative weights [64]. An improvement is expected to be detected, represented by an increase of 1 point in the autonomous subscale and a reduction of at least 1 point in the less self-determined subscale in the intervention group compared to the control group.

The Late-Life Function and Disability Instrument (LLFDI) questionnaire [65], translated and adapted for the Brazilian population [66], will be used to assess functional performance in home and community settings. This comprehensive and sensitive instrument was developed to document changes resulting from the aging process. The instrument comprises two components, disability and function, which constitute separate scales. In this study, only the disability component, which documents the frequency and limitations of individuals performing 16 activities of daily living, including basic, instrumental, and advanced activities, was used. In addition to the total scores (total frequency and total limitation), it is possible to obtain a score for each domain that comprises this scale, namely, personal role, social role, instrumental role, and management role [66]. An improvement is expected, as evidenced by a 2.4-point increase in the Z score; these data were collected one week before and one week after the intervention.

Perceived benefits and barriers to physical activity will be assessed using the EBBS-Brazil questionnaire [67], which consists of 42 items: 14 from the Barrier Scale (EBBSBAR) and 28 from the Benefits Scale (EBBSBEN). The EBBSBEN score will be calculated by summing 28 items across five domains: biological aspects, physical performance, psychological aspects, social interaction, and preventive health. The EBBSBAR score will be calculated by summing 14 items across four domains: time expenditure, physical effort, exercise environment, and family discouragement. Higher values indicate greater perceived benefits or barriers [67, 68]. An improvement is expected, as evidenced by a 2-point reduction in the score for barrier perception and a 1-point increase in the score for benefit perception. The data will be collected one week before the intervention and one week after the intervention.

Environmental perception will be assessed using the environmental perception scale for physical activity [69], which is composed of questions based on the *Neighborhood Environment Walkability Scale (NEWS)* [70, 71] and a social support scale for physical activity [72]. This version consists of 38 questions and has been validated for use with Brazilian adults [69]. This tool includes characteristics of the environment (built, natural, and social): access to commerce and places for physical activity; traffic safety; crime safety; aesthetics; neighborhood satisfaction; street and sidewalk quality; lighting; pollution; and social support for physical activity.

### Monitoring of data quality

To ensure data quality, the research assistant who collects the data sheets, also will provide feedback to the principal researcher if there is evidence that the protocol is not being followed. Data will be entered and double-checked by two people, and inconsistencies resolved by contacting the participant where appropriate or via consensus. In the event that participants discontinue or deviate from the intervention protocols, we have planned to collect specific outcome data to understand the reasons behind their decisions and assess the impact on the study results.

## Discussion

The evaluation of environmental interventions for individuals with COPD has been limited to a few rigorous studies [32–34]. This study involved a blinded, controlled clinical trial in which participants in the intervention group played an active role in selecting the most suitable walking environment. We outline the rationale and processes of a study that investigates the effectiveness of an environmental intervention involving predefined walking routes in urban areas within the neighborhood, compared to free-form walks, on physical activity and secondary outcomes, including exercise capacity, quality of life, and perceptions of dyspnea, anxiety, and depression. Additionally, we examined the barriers and benefits related to physical activity, exercise motivation, and social engagement in patients with COPD.

While moderate physical activity is highly recommended for individuals with COPD [9], symptoms, physical limitations, social barriers, and motivation often present significant challenges to adherence in this population. A personalized approach that considers environmental, social, and cultural factors to promote physical activity through tailored environmental interventions, accounting for individual differences, preferences, and needs, may enhance adherence and lead to clinical and functional benefits for individuals with COPD.

Several strengths are worth highlighting in this study protocol: i) the intervention is cost-effective and takes place in environments that reduce transportation barriers and costs by being located near the participant’s neighborhood; ii) changes in active behavior will be measured using a validated accelerometer and standardized data extraction; and iii) the protocol incorporates motivational incentive strategies for both groups by offering weekly goals for increasing walking time or the number of steps, monitored by pedometers provided to participants for eight weeks. Participants will also keep their walking and symptom diaries and receive periodic messages or calls for feedback and encouragement to continue walking. In addition, two lectures will be provided on COPD incidence information, symptom management, and the benefits of walking. Although these actions will be offered to both groups, it is possible that combining walking guidance in a favorable environment with participant preferences may result in higher levels of physical activity.

However, several potential limitations may introduce bias into the study: i) the perception of the environment, as participants become accustomed to the environment and personalized walking routes throughout the study, and their perception may change, affecting the consistency of environmental perception over time; ii) interference from external events, such as construction work or changes in the urban environment, can affect the perception of the environment during the study, introducing variability in participants’ responses; and iii) influence by the specific environment in which the intervention was carried out. Environmental conditions, such as climate, topography, and population density, can vary between different locations, limiting geographic generalization. Therefore, at the end of the study, topographical information and population density, among other characteristics in which the participants were included, were provided, allowing other researchers to evaluate the relevance of the results for different contexts.

In addition to the benefits for this clinical population, this study may offer valuable insights for public health by highlighting the role of the urban environment in promoting physical activity among COPD patients and emphasizing the importance of influencing public policies related to urban planning, transportation, and the creation of public spaces conducive to physical activity.

### Statistical analysis

All the statistical analyses were conducted using SPSS software version 22.0 (IBM Corporation, Somers, New York, USA). The normality of the data was assessed using the Shapiro–Wilk test. Variables with a normal distribution are presented as the mean and standard deviation, nonnormally distributed variables are presented as the median and interquartile range (25–75), and categorical variables are presented as the absolute and relative frequency. A significance level of 5% was used for all the statistical tests.

The data will be analyzed based on the principles of intention-to-treat, including all available data, regardless of intervention completion. Primary and secondary outcomes will be analyzed using mixed linear models [73], with analysis of ROC curve points and the minimum clinically important difference. The percentage of missing data, effect size, and other nonnormally distributed data were considered criteria for covariance structures in the mixed linear model [74, 75]. All the data will be included in the article and its supplementary materials.

## Supporting information

S1 Appendix SPIRIT 2013 checklist: Recommended items to address in a clinical trial protocol and related documents*. (DOCX)

S2 Appendix (DOCX)

S3 Appendix (DOCX)

S4 Appendix (PDF)

S1 Protocol (DOCX)

S2 Protocol (DOCX)
