# Supplementary material for: Exploring the impact of the environment on physical activity in patients with chronic obstructive pulmonary disease (EPCOT)—A comparative analysis between suggested and free walking: Protocol study
Source: PLoS One. 2024 Aug 13;19(8):e0306045. doi: 10.1371/journal.pone.0306045 (PMC11321554; doi:10.1371/journal.pone.0306045)
Supplement: S2 Appendix — (DOCX) [file pone.0306045.s002.docx]

**UNIVERSITY HOSPITAL OF THE FEDERAL UNIVERSITY OF JUIZ DE FORA**

Research Ethics Committee with

Human beings from HU-UFJF

Postgraduate program of in Rehabilitation Sciences and Physical-Functional Performance Responsible Researcher: Carla Malaguti

Address: Rua Eugênio do Nascimento, S/n - Dom Bosco

CEP: 36038-330 Juiz de Fora – MG

Telephone: (32) 99153-4633

Email: carlamalaguiti@gmail.com

**FREE AND INFORMED CONSENT TERMS**

You are being invited as a volunteer to participate in the research **“ Ecological determinants of active behavior in people with Chronic Obstructive Pulmonary Disease and effects of walking”** . In this study we intend **to investigate individual factors (age, sex, physical capacity, health status, comorbidities, motivation, anxiety and depression, quality of life and perception), interpersonal (such as social and community participation) and environmental factors (such as walkability) that may influence the physical activity of people with COPD .** The reason we study it is that physical activity is affected by several factors, the comprehensive ecological model (including interrelationships between personal factors and their physical environments) seems to explain, proposing that determinants at all levels - individual, social, environmental and politician - are taxpayers. In this sense, this project aims to investigate individual factors (age, sex, physical capacity, health status, comorbidities, motivation and perception), interpersonal factors (such as social and community participation) and environmental factors (such as walkability), which can influence the physical activity of people with COPD and should be considered to provide support for designing better adherence strategies for physical activity in this population. Secondarily, we will carry out a controlled clinical trial, that is, a study in which two groups will be compared in a "double blind" manner, in which neither you nor the evaluator who will apply the tests will know which group you will be in. Right at the beginning of the study, a draw will be held to decide which group you will belong to. The two groups are very similar, the exception is some type of orientation. However, at the end of the study, if it is proven that the group you belonged to did not have greater benefits compared to the other group, you will be invited to participate in this other group, if you wish.

For this study we will adopt the following procedures: We will measure the level of physical activity using the Actigraph GT3X® accelerometer . Using an elastic strap, the device will be fixed at the waist level of the lower limb, and will only need to be removed when taking a shower, carrying out water activities and while sleeping. The device has a minimum size and dimensions that do not affect comfort during use, A manual will be offered containing information and instructions on how to use the device, and a diary to fill out about the day of the week and the times to attach and remove the device. You must wear the accelerometer for at least 4 days, including one weekend day. We will carry out a mental function test, which is a simple questionnaire to assess your understanding of the year, week and month and memorization of some words. Lung function will be assessed through spirometry, we will perform 3 measurements of maximum forced expiration and the data will be expressed in absolute values and percentage of predicted for the Brazilian population . We will apply questionnaires and/or scales to assess dyspnea, impact of COPD symptoms on daily life, perceived barriers to physical activity, quality of life using a specific questionnaire for respiratory diseases, anxiety and depression, social participation , motivation for exercise, and walkability. We will perform the Six-Minute Walk Test (6MWT) , which will be done in a 30-meter long corridor with a smooth surface, where participants will be instructed to walk as far as possible during the six minutes. The participant will be allowed to rest if necessary, but the timer will not be stopped. Two tests will be carried out with a 30-minute rest interval between them. Heart rate and pulse hemoglobin saturation (SpO _2_ ) will be continuously monitored. Systemic blood pressure, symptoms of dyspnea and fatigue will also be measured using the modified Borg scale before and after the test. The test may be interrupted by the participant or the evaluator if there is malaise, nausea, severe dyspnea, extreme fatigue, chest pain, headache or if SpO _2_ ≤ 85%. The Charlson index will be used to assess the presence of comorbidities, as it helps professionals classify patients based on the severity of the diseases. The risks involved in the research consist of muscle fatigue, dyspnea, coughing fits, muscle pain, reduced oxygen levels, risk of falling due to the tests and embarrassment when answering the questionnaires. To reduce the chance of these risks occurring, the examining physiotherapist will ask you throughout the tests how your level of fatigue is and, if necessary, the effort will be interrupted for you to rest. The examiner will be monitoring your heart rate and blood oxygen level with a simple device called a pulse oximeter placed on one of your fingers, and if necessary, the tests will be stopped and oxygen may be offered if necessary . The questionnaires and scales will be answered in a private place and with freedom not to answer questions that they consider embarrassing. The research will contribute to “direct and/or indirect research benefits”. The results of this research can help to identify your physical capacity, and from this, appropriate physiotherapeutic treatments can be offered according to your level of physical capacity so that you can increasingly improve your physical condition to the point of providing independence. , that is, that you can carry out everyday activities without needing help . And the change in the urban environment, with the development of a more inclusive, safe, sustainable and resilient city.

There will be no cost to participating in this study, nor will you receive any financial advantage. Despite this, if damages are identified and proven resulting from this research, you are guaranteed the right to compensation. You will be informed about the study in any aspect you wish and will be free to participate or refuse to participate. You may withdraw your consent or stop participation at any time. Your participation is voluntary and refusal to participate will not result in any penalty or change in the way you are served by the researcher, who will treat your identity with professional standards of confidentiality , in compliance with legislation. Brazilian (Resolution No. 466/12 of the National Health Council), using the information only for academic and scientific purposes.

The search results will be available to you when completed. Your name or material indicating your participation will not be released without your permission. You will not be identified in any publication that may result from this study. The data and instruments used in the research will be archived with the responsible researcher for a period of 5 (five) years, and after this time they will be destroyed. This consent form is printed in two original copies, one copy will be filed by the responsible researcher, at the **“Faculdade de Fisioterapia da UFJF” Center** and the other will be provided to you.

I, ____________________________________________, holder of the ____________________ Identity document, was informed of the objectives of the study **“ Ecological determinants of the active behavior of people with Chronic Obstructive Pulmonary Disease and effects of walking”** , in a clear and detailed manner and clarified my doubts. I know that at any time I can request new information and change my decision to participate if I wish.

I declare that I agree to participate in this study. I received a copy of this informed consent form and was given the opportunity to read it and clarify my doubts.

Juiz de Fora, _________ of __________________________ of ______.

__________________________________________ ____________________

signature of the participant Date

__________________________________________ ____________________

Name and signature of the researcher Date

__________________________________________ ____________________

Name and signature of witness Date

If you have any doubts regarding the ethical aspects of this study, you can consult the HU-UFJF Research Ethics Committee:

Rua Catulo Breviglieri , s/nº - Bairro Santa Catarina
CEP.: 36036-110 - Juiz de Fora – MG

Telephone: 4009-5167

Email: cep.hu@ufjf.edu.br
