# Supplementary material for: Exploring the impact of the environment on physical activity in patients with chronic obstructive pulmonary disease (EPCOT)—A comparative analysis between suggested and free walking: Protocol study
Source: PLoS One. 2024 Aug 13;19(8):e0306045. doi: 10.1371/journal.pone.0306045 (PMC11321554; doi:10.1371/journal.pone.0306045)
Supplement: S3 Appendix — (DOCX) [file pone.0306045.s003.docx]

**HOSPITAL UNIVERSITÁRIO DA UNIVERSIDADE FEDERAL DE JUIZ DE FORA**

Comitê de Ética em Pesquisa com

Seres Humanos do HU-UFJF

Mestrado em Ciências da Reabilitação e Desempenho Físico-Funcional Pesquisador Responsável: Carla Malaguti Endereço: Rua Eugênio do Nascimento, S/n - Dom Bosco CEP: 36038-330 Juiz de Fora – MG Telefone: (32) 99153-4633 E-mail: carlamalaguiti@gmail.com

**TERMO DE CONSENTIMENTO LIVRE E ESCLARECIDO**

O Senhor (a) está sendo convidado (a) como voluntário (a) a participar da pesquisa **“Determinantes ecológicos do comportamento ativo de pessoas com Doença Pulmonar Obstrutiva Crônica e efeitos da caminhabilidade”**. Neste estudo pretendemos **investigar fatores individuais (idade, sexo, capacidade física, estado de saúde, comorbidades, motivação, ansiedade e depressão, qualidade de vida e percepção), interpessoais (como participação social e comunitária) e fatores ambientais (como a caminhabilidade) que possam inﬂuenciar a atividade física de pessoas com DPOC.** O motivo que nos leva a estudar é que a atividade física é afetada por diversos fatores, o modelo ecológico (incluindo interrelações entre fatores pessoais e seus ambientes físicos) abrangente parece explicar, propondo que determinantes em todos os níveis - individual, social, ambiental e político - são contribuintes. Nesse sentido, esse projeto visa investigar desde os fatores individuais (idade, sexo, capacidade física, estado de saúde, comorbidades, motivação e percepção), fatores interpessoais (como participação social e comunitária) e fatores ambientais (como a caminhabilidade), que possam inﬂuenciar a atividade física de pessoas com DPOC e que deve ser considerada para trazer subsídios para traçar estratégias de melhor adesão para atividade física dessa população. Secundariamente, realizaremos um ensaio clínico controlado, ou seja, um estudo no qual serão comparados dois grupos de forma "duplo cega", no qual nem você nem o avaliador que aplicará os testes saberão em qual grupo você estará. Pois logo no início do estudo será feito um sorteio para definir para qual grupo você pertencerá. Os dois grupos são muito parecidos, a exceção é quanto a algum tipo de orientação. Porém, no final do estudo se ficar comprovado que o grupo que você pertenceu não teve maiores benefícios comparado ao outro grupo, você será convidado a participar deste outro grupo, se você desejar.

Para este estudo adotaremos os seguintes procedimentos: Iremos realizar a medida do nível de atividade física por meio do acelerômetro Actigraph GT3X®. Através de uma cinta elástica o dispositivo ficará fixo no nível da cintura de membro inferior, sendo necessário retirá-lo apenas ao tomar banho, realizar atividades aquáticas e durante o sono. O dispositivo tem tamanho e dimensões mínimas que não afetam o conforto durante sua utilização, será ofertado um manual contendo as informações e instruções sobre o uso do dispositivo, e um diário para preenchimento sobre o dia da semana e os horários de fixação e de retirada do dispositivo. É necessário usar o acelerômetro por pelo menos 4 dias, incluindo um dia do fim de semana. Realizaremos um teste de função mental, que é um questionário simples para avaliar seu entendimento em relação ao ano, semana e mês e memorização de algumas palavras. Será avaliado a função pulmonar através da espirometria, realizaremos 3 medidas da expiração máxima forçada e os dados serão expressos em valores absolutos e porcentagem do previsto para população brasileira. Iremos aplicar questionários e/ou escalas para avaliação da dispneia, impacto dos sintomas da DPOC no dia a dia, barreiras percebidas para atividade física, qualidade de vida por um questionário específico para doenças respiratórias, ansiedade e depressão, participação social, , motivação para o exercício, e caminhabilidade. Realizaremos o Teste de Caminhada de Seis Minutos (TC6), que será feito em um corredor com 30 metros de comprimento e com superfície lisa, onde os participantes serão instruídos a caminhar a maior distância possível durante os seis minutos. Será permitido ao participante descansar caso este necessite, porém o cronômetro não será interrompido. Serão realizados dois testes com um intervalo de 30 minutos de repouso entre eles. A frequência cardíaca e a saturação da hemoglobina de pulso (SpO_2_) serão continuamente monitorizadas. Serão mensuradas também a pressão arterial sistêmica, os sintomas de dispneia e a fadiga pela escala de Borg modificada antes e após o teste. O teste poderá ser interrompido pelo próprio participante ou pelo avaliador caso haja mal-estar, náuseas, dispneia importante, fadiga extrema, precordialgia, cefaleia ou se SpO_2_ ≤ 85%. O índice de Charlson será utilizado para avaliar a presença de comorbidades, pois auxilia os profissionais a classificar pacientes baseando-se na gravidade das doenças. Os riscos envolvidos na pesquisa consistem em cansaço muscular, dispneia, crise de tosse, dor muscular, redução dos níveis de oxigênio, risco de queda decorrente dos testes e constrangimento ao responder os questionários. Para diminuir a chance desses riscos acontecerem, o fisioterapeuta examinador estará perguntado a você durante toda a realização dos testes como está seu nível de cansaço e se necessário for o esforço será interrompido para seu descanso. O examinador estará monitorando sua frequência cardíaca e nível de oxigênio no sangue com um aparelho simples chamado oxímetro de pulso colocado em um dos dedos da mão, e se caso necessário for, os testes serão interrompidos e oxigênio pode ser oferecido se necessário. Os questionários e escalas serão respondidos em um local reservado e com liberdade para não responder questões que julgar constrangedoras. A pesquisa contribuirá para “benefícios da pesquisa diretos e/ou indiretos”. Os resultados dessa pesquisa poderão ajudar na identificação de como está sua capacidade física, e a partir disso poderão ser oferecidos tratamentos fisioterapêuticos apropriados de acordo com seu nível de capacidade física para que se possa melhorar sua condição física cada vez mais a ponto de proporcionar a independência, ou seja, que você possa realizar atividades do dia a dia sem precisar de ajuda. E a mudança no ambiente urbano, com desenvolvimento de uma cidade mais inclusiva, segura, sustentável e resiliente.

Para participar deste estudo você não terá nenhum custo, nem receberá qualquer vantagem financeira. Apesar disso, caso sejam identificados e comprovados danos provenientes desta pesquisa, o Senhor (a) tem assegurado o direito a indenização. O Sr. (a) será esclarecido (a) sobre o estudo em qualquer aspecto que desejar e estará livre para participar ou recusar-se a participar. Poderá retirar seu consentimento ou interromper a participação a qualquer momento. A sua participação é voluntária e a recusa em participar não acarretará qualquer penalidade ou modificação na forma em que o Sr. (a) é atendido (a) é atendido pelo pesquisador, que tratará a sua identidade com padrões profissionais de sigilo, atendendo a legislação brasileira (Resolução Nº 466/12 do Conselho Nacional de Saúde), utilizando as informações somente para os fins acadêmicos e científicos.

Os resultados da pesquisa estarão à sua disposição quando finalizada. Seu nome ou o material que indique sua participação não será liberado sem a sua permissão. O (A) Senhor (a) não será identificado (a) em nenhuma publicação que possa resultar deste estudo. Os dados e instrumentos utilizados na pesquisa ficarão arquivados com o pesquisador responsável por um período de 5 (cinco) anos, e após esse tempo serão destruídos. Este termo de consentimento encontra-se impresso em duas vias originais, sendo que uma via será arquivada pelo pesquisador responsável, no Centro **“Faculdade de Fisioterapia da UFJF”** e a outra será fornecida ao Senhor (a).

Eu, ____________________________________________, portador do documento de Identidade ____________________ fui informado (a) dos objetivos do estudo **“Determinantes ecológicos do comportamento ativo de pessoas com Doença Pulmonar Obstrutiva Crônica e efeitos da caminhabilidade”**, de maneira clara e detalhada e esclareci minhas dúvidas. Sei que a qualquer momento poderei solicitar novas informações e modificar minha decisão de participar se assim o desejar.

Declaro que concordo em participar desse estudo. Recebi uma via deste termo de consentimento livre e esclarecido e me foi dada à oportunidade de ler e esclarecer as minhas dúvidas.

Juiz de Fora, _________ de __________________________ de ______.

 __________________________________________ ____________________

Nome e assinatura do (a) participante (a) Data

 __________________________________________ ____________________

Nome e assinatura do (a) pesquisador (a) Data

 __________________________________________ ____________________

Nome e assinatura da testemunha Data

Em caso de dúvidas com respeito aos aspectos éticos deste estudo, você poderá consultar o Comitê de Ética em Pesquisa HU-UFJF:

Rua Catulo Breviglieri, s/nº - Bairro Santa Catarina
CEP.: 36036-110 - Juiz de Fora – MG

Telefone: 4009-5167

E-mail: cep.hu@ufjf.edu.br
