# Supplementary material for: Exploring the impact of the environment on physical activity in patients with chronic obstructive pulmonary disease (EPCOT)—A comparative analysis between suggested and free walking: Protocol study
Source: PLoS One. 2024 Aug 13;19(8):e0306045. doi: 10.1371/journal.pone.0306045 (PMC11321554; doi:10.1371/journal.pone.0306045)
Supplement: S4 Appendix — (PDF) [file pone.0306045.s004.pdf]

RECOMMENDATIONS FOR WALKING

The **American Heart Association** recommends a **minimum** of **150 minutes** (2 hours and 30 minutes) of **moderate-intensity** physical activity or **75** minutes of **high-intensity** exercise per week.

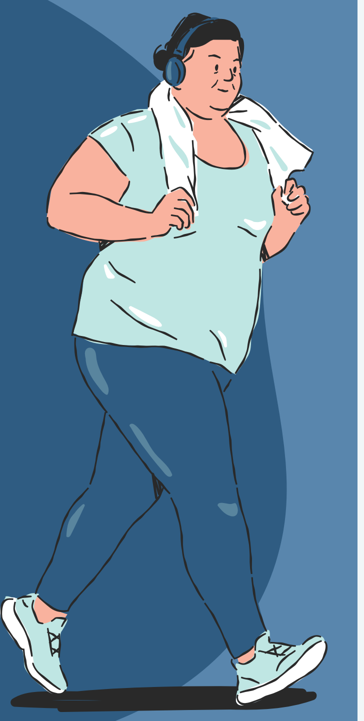

So, **30 minutes** of moderate-paced walking, **five times a week**, is what you need to attain several **quality-of-life-related benefits**.

BENEFITS OF WALKING

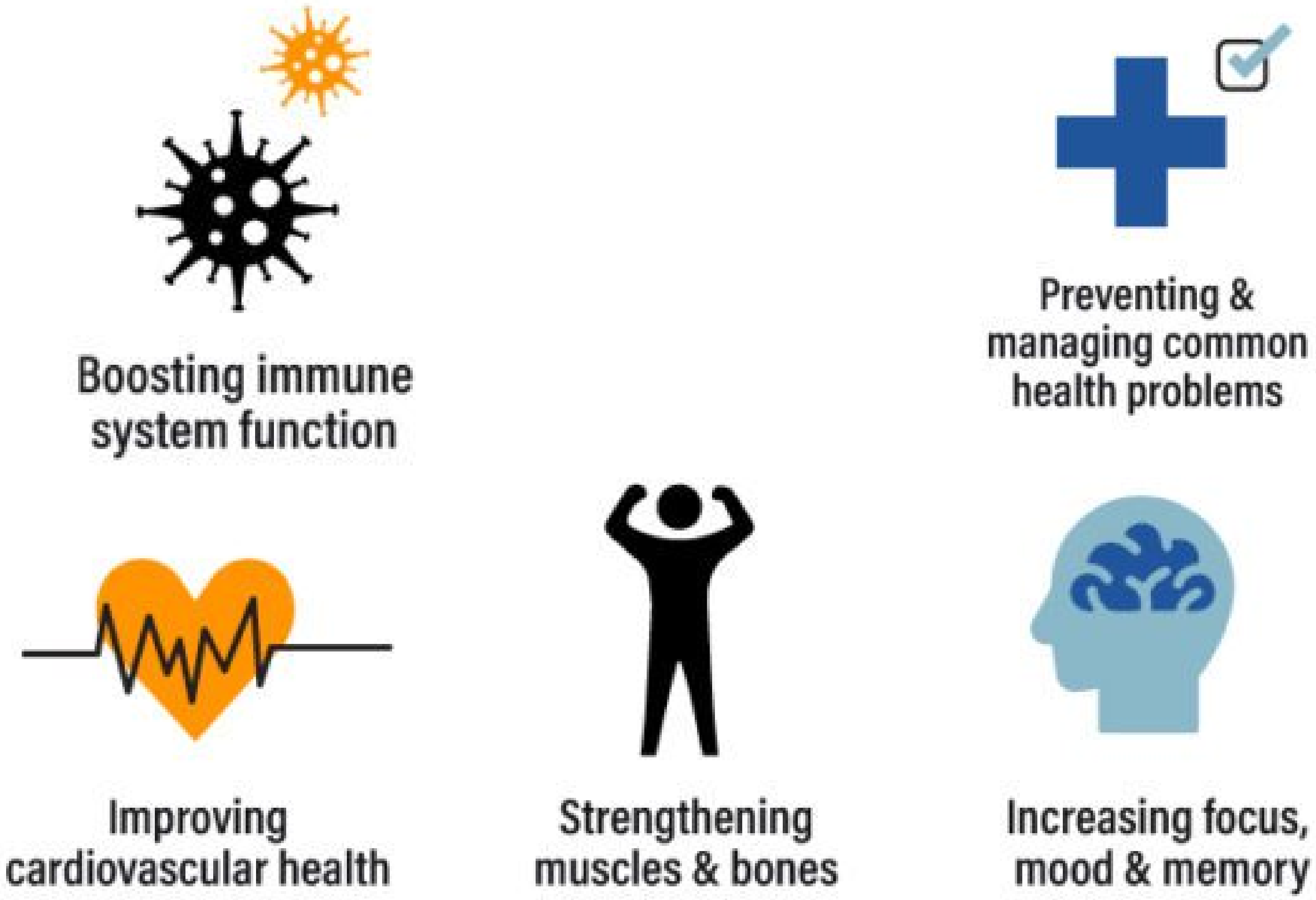

RECOMMENDATIONS

1. Do not exercise when feeling unwell, experiencing excessive shortness of breath, or extreme fatigue.
2. Perform exercises every day.
3. Have a light meal before exercising.
4. Wear appropriate shoes with good cushioning.
5. Wear comfortable clothing.
6. Drink water before, during, and after the walk.
7. Apply sunscreen.
8. Do stretches before and after the walk, such as stretching your legs and arms, to improve circulation and prevent cramps

STRETCHING

"Whenever you stretch one leg, do the same with the other before moving to another muscle, holding each position for 20 seconds."

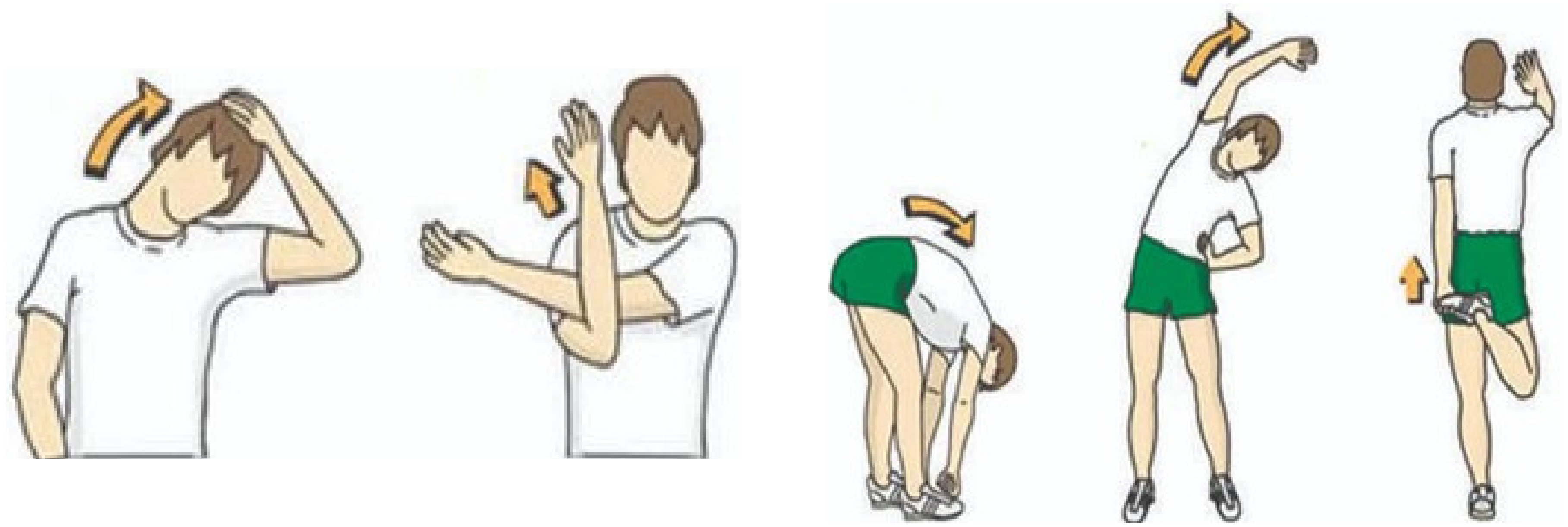

"When performing the stretch, you should not feel pain, only the muscle stretching.

Start by walking slowly, and only after 5 minutes, increase the walking pace. In the last 10 minutes of the walk, decrease the pace."

POSTURE

Maintain a posture that is as natural as possible, keeping your spine upright, and ensuring that your feet make contact with the ground in a aligned manner, without turning inwards or outwards. Keep your arms slightly bent to assist with balance.

Breathe correctly: Inhale through your nose (inspiration) and exhale through your mouth (expiration).
When the effort becomes significant, inhale and exhale through your mouth.

POSTURE

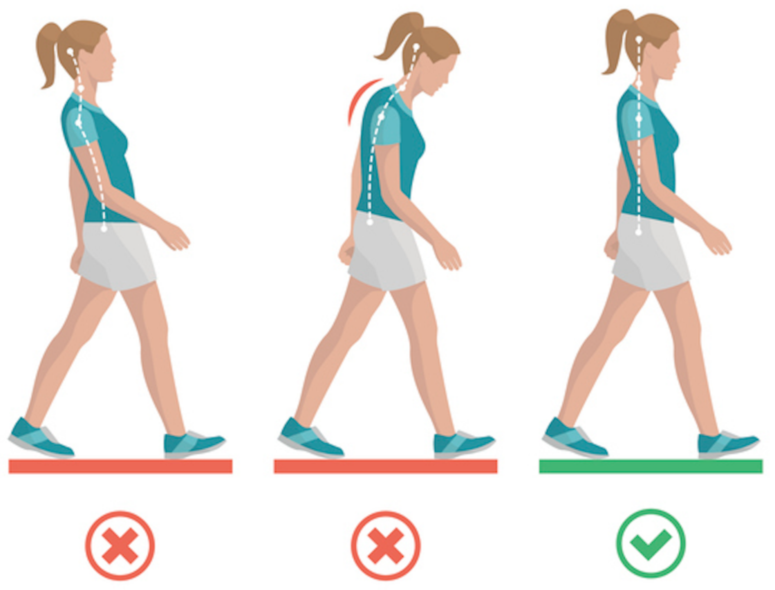

increase the walking time progressively.

1ST WEEK:
3 TIMES A WEEK FOR 15 MINUTES EACH TIME, TOTALING 45 MINUTES PER WEEK.
5 TIMES A WEEK FOR 15 MINUTES EACH TIME, TOTALING 75 MINUTES PER WEEK.

2ST WEEK:
3 TIMES A WEEK FOR 20 MINUTES EACH TIME, TOTALING 60 MINUTES PER WEEK.
5 TIMES A WEEK FOR 20 MINUTES EACH TIME, TOTALING 100 MINUTES PER WEEK.

3ST WEEK:
3 TIMES A WEEK FOR 30 MINUTES EACH TIME, TOTALING 90 MINUTES PER WEEK.
5 TIMES A WEEK FOR 30 MINUTES EACH TIME, TOTALING 150 MINUTES PER WEEK.

4ST WEEK:
3 TIMES A WEEK FOR 40 MINUTES EACH TIME, TOTALING 120 MINUTES PER WEEK.
5 TIMES A WEEK FOR 40 MINUTES EACH TIME, TOTALING 200 MINUTES PER WEEK.

5ST WEEK:
3 TIMES A WEEK FOR 50 MINUTES EACH TIME, TOTALING 150 MINUTES PER WEEK.
5 TIMES A WEEK FOR 50 MINUTES EACH TIME, TOTALING 250 MINUTES PER WEEK.
