# Supplementary material for: Exploring the impact of the environment on physical activity in patients with chronic obstructive pulmonary disease (EPCOT)—A comparative analysis between suggested and free walking: Protocol study
Source: PLoS One. 2024 Aug 13;19(8):e0306045. doi: 10.1371/journal.pone.0306045 (PMC11321554; doi:10.1371/journal.pone.0306045)
Supplement: S1 Protocol — (DOCX) [file pone.0306045.s005.docx]

**UNIVERSITY HOSPITAL OF THE FEDERAL UNIVERSITY OF JUIZ DE FORA**

## Human Research Ethics Committee from HU-UFJF

## *Research Ethics Committee HU - UFJF*

Project and Project Coordinator Data

| **Project title** | *Ecological determinants of active behavior in people with Chronic Obstructive Pulmonary Disease and effects of walkability* |
| --- | --- |
| **Researchers** | Larissa Guimarães Paiva  Nara Batista de Souza  Túlio Medina Dutra de Oliveira  Anderson José  Carla Malaguti |
| **Contact:** |  |
| **Unit/Department:** | Faculty of Physiotherapy / Cardiorespiratory and Musculoskeletal Physiotherapy Department |
| **Sector/Unit where the research will be carried out in**  **HU-UFJF/Ebserh (when applicable):** | Multidisciplinary Unit/Cardiopulmonary Rehabilitation Outpatient Clinic |
| **Date:** | 01/04/2023 |

***1.* Justification/Characterization of the Problem**

Physical inactivity is a common feature of many chronic diseases, both as a cause and a consequence. The high prevalence of physical inactivity is a problem ^1,2^ that contributes to increased morbidity, higher rates of premature death ^3^ , and increased costs ^4^ . The World Health Organization (WHO) ^5^ , the American College Sports of Medicine ^6^ , as well as the Physical Activity Guide for the Brazilian Population ^7^ recommend accumulating 150 minutes of moderate physical activity per week, or 75 minutes of vigorous physical activity, which translates into health-related benefits. Furthermore, it is also recommended to accumulate 7,500 to 10,000 steps per day for adults ^8^ , and around 5,000 steps per day for the elderly and special populations ^9^ , such as in chronic lung diseases. In patients with chronic obstructive pulmonary disease (COPD), signiﬁcantly lower levels of physical activity were observed compared to healthy controls ^10-12^ . Existing data shows that time spent walking is shorter in COPD patients compared to healthy people of the same age ^11,13,14^ . Associations have also been observed between physical activity and clinical characteristics of patients with COPD, such as disease severity, comorbidities, exacerbations and behavioral factors ^15^ . Prospective longitudinal studies have shown an association between low levels of physical activity and an increased number of exacerbations, risk of hospitalizations and death from all causes in patients with COPD ^16-17^ . Studies have focused on testing the effectiveness of interventions such as pulmonary rehabilitation programs, pharmacological treatment, oxygen, as well as behavioral interventions to increase physical activity in patients with COPD ^18-19^ . However, these interventions have focused only on the level of individual factors, and have not resulted in lasting changes to a more active lifestyle in this population ^19^ . On the other hand, it must be recognized that, in general, physical activity depends on many factors, in addition to biological, behavioral and genetic factors, as well as social, environmental, cultural and public policy factors. The ecological model takes a broad view of the cause of inactive behavior, with the social factor and the environment also included as contributors, particularly external to the health factor, such as areas of urban planning, transport systems and open public spaces such as squares, parks and ^20 cycle paths^ . The urban environment has the potential to contribute substantially to physical activity. It has already been demonstrated that living in physical activity-friendly cities can help residents achieve around 45-59% of the 150min/week recommended guidelines for physical activity ^21^ . Walkability refers to the extent to which an area is suitable for walking and active transport ^22^ . Walkability has been the subject of increasing research in the health sector, including different populations such as the elderly ^23^ , children and adolescents ^24^ , and conditions such as diabetes risk ^25^ , obesity ^25,26^ and cardiovascular risk ^27,28^ . However, the ecological model involving the environmental factor as a determinant of physical activity in COPD has not yet been investigated. Because physical activity is affected by many factors, the overarching ecological model (including interrelationships between personal factors and their physical environments) appears to explain physical activity, proposing that determinants at all levels—individual, social, environmental, and political—are contributors. In this sense, this project aims to investigate individual factors (age, sex, physical capacity, health status, comorbidities, motivation and perception), interpersonal factors (such as social and community participation) and environmental factors (such as walkability), which can influence the physical activity of people with COPD and should be considered to provide support for designing better adherence strategies for physical activity in this population. Secondarily, a controlled clinical trial comparing a group walking guided on routes with better walkability with a group walking on a free route, could provide information on the environmental impact on adherence to physical activity in this population.

**2.Objectives**

**2.1 General**

The objective of this study is to investigate individual factors (age, sex, physical capacity, health status, comorbidities, motivation, anxiety and depression, quality of life and perception), interpersonal factors (such as social and community participation) and environmental factors (such as walkability) that may influence the physical activity of people with COPD. When identifying routes with better walkability, carry out a controlled clinical trial comparing a group walking guided along pre-established routes with better walkability with a group walking on a free route.

**2.2 Specifics**

1: Evaluate individual indicators: age, sex, severity of the disease, physical capacity, health status, comorbidities, motivation, self-efficacy for exercise, levels of anxiety and depression, quality of life, perception of barriers and benefits for activity physical and level of physical activity measured objectively using an accelerometer; evaluate interpersonal indicators through social and community participation; evaluate environmental indicators through walkability in the participants' community. 2: Identify possible relationships and determining factors for physical activity in patients with COPD. 3: Carry out an educational intervention enhancing facilities and dissolving barriers, involving topics such as clinical control of the disease, empowerment to develop self-efficacy for physical activity, benefits of exercise, prescription of guided walking with or without a pre-established route, techniques for energy conservation to control symptoms, exercise guidelines to reduce the risk of falling, and motivational strategies in search of active behavior for study participants. 4: Evaluate the effectiveness of guided walking on routes or trails with better walkability compared to walking on a free route, in terms of adherence to the practice of physical activity in the community, as well as its effects on personal and interpersonal indicators. 5: Prepare reports for the health and urban planning department of the city of Juiz de Fora, as well as for the local media, in order to raise awareness of the development of actions to achieve a friendly city, with more walkable areas, favorable to practice of safe physical activity for everyone.

**3.Methodology and Action Strategies**

**Study Design**

This is a prospective study to be conducted from the date of approval by the Research Ethics Committee of the University Hospital of the Federal University of Juiz de Fora. All patients who agree to participate in the study will sign the Informed Consent Form (TCLE). Individuals will be recruited from the list of patients diagnosed with COPD referred to the physiotherapy and pulmonary rehabilitation service at the University Hospital of the Federal University of Juiz de Fora and Primary Health Care Units in the city of Juiz de Fora. Everyone will undergo an interview and assessment of multilevel factors for the practice of physical activity of patients with COPD to obtain their determinants (Stage 1), and with a subsequent invitation to the controlled clinical trial (Stage 2) with guidelines for physical activity through walks with guided routes with better walkability (experimental group) compared to free walking guidance (control group).

**Participants**

Individuals with a confirmed diagnosis of COPD will be studied based on the *Global Initiative for Chronic Obstructive Lung Disease* (GOLD) criteria, with the relationship between post-bronchodilator forced expiratory volume in the first second (FEV _1_ )/forced vital capacity (FVC) being < 70% and FEV _1_ < 80% of the predicted value ^29^ , of both sexes, aged over 18 years, clinically stable, without unstable cardiovascular diseases, neurological or musculoskeletal disorders and able to carry out the proposed evaluations and interventions. Participants who are unable to perform or understand the study assessments will be excluded from the study due to physical or psychological disability and a primary diagnosis of a respiratory disease other than COPD, supported by oxygen therapy, with orthopedic and/or neurological limitations that prevent them from performing the tasks. tests, recent hospitalization (last 3 months), cognitive impairment identified by a score less than or equal to 4 on the *Six Item Screener* ^30,31^, or who expressed their desire to withdraw from the study.

**Procedures and measures:**

In Stage 1, individual data will be obtained such as clinical-demographic information, severity of the disease, comorbidities, exercise capacity, level of physical activity, activities of daily living, anxiety and depression, risk and fear of falling, quality of life, motivation and self-efficacy for exercise, as well as barriers and benefits to physical activity. Interpersonal information will also be obtained through instruments of social participation, community participation; in addition to environmental information such as walkability measurements. These assessments will be carried out in two visits to avoid consuming time and fatigue for the participant. In Stage 2, after assessments and analysis to determine the factors that impact the physical activity of this population, patients will be randomly allocated to guided walking groups on a route with better walkability or to a guided walking group without suggested route. The intervention period will last 8 weeks, with suggestions for walking 3-5 times a week and providing a diary to record the walks. All participants will receive educational interventions through lectures and printed material. Indirect supervision of both groups will be through bi-weekly phone calls or text messages. After completion of the intervention period, reassessment will be carried out. Global cognitive function will be assessed using the *Six-item Screener* which assesses recall of 3 items and orientation to year, month and day of the week, producing a score of 0 to 6 questions answered correctly. A score ≤ 4 is considered positive for cognitive impairment ^30,31^ and will indicate exclusion of the participant from the protocol.

**Sample characterization variables:**

**Lung Function**

Spirometry will be performed using a portable spirometer (Spirobank II, Medical International Research, New Berlin, USA). The technical procedures, acceptability and reproducibility criteria adopted will be determined in accordance with the Guidelines for pulmonary function testing ^32^. The maneuver will be explained and demonstrated in practice by previously trained researchers. The participant will be asked to perform three reproducible forced maximum expiration maneuvers, sustaining the expiration during the period of presence of expiratory flow. The data will be expressed in absolute values and percentage of predicted for the Brazilian population ^33^.

**Impact of Symptoms**

The impact of COPD symptoms will be assessed using the *COPD Assessment Test* (CAT) Questionnaire, an instrument for quantifying the impact of COPD symptoms on daily life. The CAT is made up of eight items, called cough, expectoration, chest tightness, shortness of breath, limitations in home activities, confidence in leaving the house, sleep and energy. For each item, the patient chooses only one answer option, whose score varies from zero to five. The results vary according to the range of scores obtained, classified in relation to the clinical impact: 6-10 points: mild; 11-20: moderate; 21-30: severe; 31-40: very serious ^34^.

**Comorbidities**

The Charlson index will be used to assess the presence of comorbidities. This instrument has been widely used in research to identify medical conditions that are not found in medical records. In the context of clinical practice, the ICC helps professionals classify patients based on the severity of diseases, creating appropriate resource allocation schemes. The index is made up of 19 clinical conditions, selected based on their effect on the patient's prognosis in terms of mortality. For each of the clinical conditions, a score is established based on relative risk and with weights ranging from 0 to 6 ^35^.

**Primary Outcome:**

The primary outcome will be through objective assessment of the level of physical activity, which will be assessed by the Actigraph accelerometer.

**Physical Activity Level**

An objective assessment of the participant's physical activity level will be carried out using the Actigraph GT3X® accelerometer (Actigraph LLC, USA), a validated and reliable physical activity monitor for use in individuals with COPD ^36^. Participants will wear an elastic strap that fixes the device at the waist level of the dominant lower limb, and will be instructed to only remove it when taking a shower, performing water activities and while sleeping. The device has a minimum size and dimensions that do not affect comfort during use, Each participant will be offered a manual containing information and instructions on how to use the device, and a diary to fill out about the day of the week and the times to attach and remove the device. For physical activity data to be valid for analysis, participants need to wear the accelerometer for at least 4 days, covering at least one weekend day ^37,38^. A day of data will be included if there is at least 10 hours of usage in a 24 hour period (0:00-23:59 hours) ^39^. Only waking periods will be retained in the analysis. The standard conversion of 1 MET = 3.5 mL/kg/min will be used and then coded into one of four absolute intensity categories: sedentary (<1.5 METs), light (1.5–2.99 METs), moderate (3.00–5.99 METs), or vigorous activity (>6 METs). Average daily PA will be calculated as total physical activity time (of all physical activity intensities) divided by the number of eligible days. Physical activity time will also be stratified according to intensity (i.e., light intensity and moderate-vigorous intensity) and sedentary behavior for analysis ^40,41^.

**Secondary Outcomes:**

**Dyspnea**

Patients will report their dyspnea according to the modified Medical Research Council scale ^42^, previously translated, culturally adapted and validated for the Brazilian population. This scale is made up of five activities whose dyspnea rating varies from 0 to 4. The higher the score, the greater the dyspnea symptom reported.

**Exercise capacity**

Exercise capacity will be assessed by the Six-Minute Walk Test (6MWT) which is considered a submaximal test used as a single measure of functional status. It will be carried out in accordance with international guidelines for the application of the test ^43^, in a corridor 30 meters long and with a smooth surface, where participants will be instructed to walk as far as possible during the six minutes. Every minute, the examiner will inform participants of the time left to complete the six minutes and will issue standardized encouraging phrases. ^43^ The participant will be allowed to rest if necessary, but the timer will not be stopped. Two tests will be carried out with a 30-minute rest interval between them. The test with the longest distance covered will be considered for analysis. Heart rate and pulse hemoglobin saturation (SpO _2_) will be continuously monitored. Systemic blood pressure, symptoms of dyspnea and fatigue will also be measured using the modified Borg scale ^44^ before and after the test. The test may be interrupted by the participant or the evaluator if there is malaise, nausea, severe dyspnea, extreme fatigue, chest pain, headache or if SpO _2_ <85%.

**Perceived barriers to physical activity**

Perceived benefits and barriers to physical activity will be assessed with the EBBS Brasil, a 42-item questionnaire: 14 belonging to the Barrier Scale (EBBSBAR) and 28 to the Benefits Scale (EBBSBEN). The EBBSBEN score was calculated by adding 28 items in five domains: biological aspects, physical performance, psychological aspects, social interaction and preventive health. The EBBSBAR score was calculated by summing 14 items in four domains: time expenditure, physical effort, exercise environment and family discouragement. Higher values indicated greater perceived benefits or barriers ^45,46.^

**Health-Related Quality of Life**

Quality of life will be assessed using the specific questionnaire for respiratory diseases Saint

George's Respiratory Questionnaire (SGRQ), which has already been translated, culturally adapted and validated for the Brazilian population ^47^. The instrument addresses aspects related to three domains:

symptoms, activity and psychosocial impacts that the respiratory disease inflicts on the patient. Each domain has a maximum possible score and its total score and for each domain it varies from 0 to 100, the lower the value, the better the quality of life.

**Anxiety and Depression**

Anxiety and depression will be measured using the Hospital Anxiety and Depression Scale (HADS) . This scale was developed to estimate the prevalence of anxiety and depression in adults and is currently also used in patients with chronic lung diseases. ^48^ The scale aims to identify cases (possible or probable) of anxiety disorders and/or depression. It consists of 14 items divided into two subscales: seven questions for the diagnosis of Anxiety Disorder and another seven for Depressive Disorder. The response scale varies between zero and three points (from absent to very frequent) with a maximum score of 21 points per subscale. Higher scores indicate greater severity of anxiety and depression ^48^.

**Social Participation**

^49.50^ questionnaire will be used is a comprehensive and sensitive instrument developed for documenting changes resulting from the aging process, it allows capturing the person's functional performance in the home and community environments . The instrument has two components: Disability and Function, which constitute distinct scales. Only the first, Disability, will be used, which documents the individual's frequency and limitations in carrying out 16 activities of daily living, comprising basic, instrumental and advanced activities. In addition to the total scores (Total frequency and Total limitation), it is possible to obtain a score for each domain that makes up this scale, namely, Personal role, Social role, Instrumental role and Management role.

**Exercise Motivation**

The Behavioral Regulation Exercise Questionnaire (BREQ) will be used to assess self-determined behavior patterns when practicing physical exercise. It was created in 1997 ^59^ and adapted into two other versions. The most current version is the BREQ-3 ^51,52^, it is an instrument translated and adapted to the Portuguese language ^53^ , it has 23 items preceded by the statement “Why do you practice physical exercise?” answered on a 5-point Likert scale, in which the respondent indicates the degree of agreement that best suits their case , ranging from “it is not true for me” (0) to “it is often true for me”(4) . These 23 items are organized into 6 domains: motivation, external regulation, introjected, identified, integrated (extrinsic motivation) and intrinsic regulation (intrinsic motivation), the six subscales that make up the BREQ-3 allow the analysis of the motivation profile for practice of physical exercise through the so-called Self-Determination Index (IaD) where different weights are assigned to each subscale, with the autonomous subscales receiving positive weights and the less self-determined subscales receiving negative weights.

**Walkability**

Walkability will be collected using the environmental perception scale for the practice of physical activity ^54^ , composed of questions based on the NEWS scale ^55,56^ and a scale of social support for the practice of physical activity ^57^ . The version consisting of 38 questions was validated for Brazilian adults ^54^ . This tool includes characteristics of the environment (built, natural and social): access to commerce and PA areas, traffic safety, safety from crime, neighborhood aesthetics and satisfaction, quality of streets and sidewalks, lighting, pollution and social support for physical activity.

**Data analysis**

The data will be analyzed using the SPSS v.22.0 program.

**4. Results and expected impacts**

It is expected that with the development of this proposal, multilevel indicators will be identified, in order to enhance facilities and dissolve barriers through educational and physical activity interventions through guided walking, aimed at promoting physical activity for people with COPD, translating into a more active clinical population. Produce reports with urban indicators that impact the promotion of physical activity and health and disease prevention for the municipality's health and urban planning departments, as well as content for dissemination in local media.

**5. Risks and benefits**

The risks and discomforts offered will be those arising from the tests, consisting of muscle fatigue, dyspnea, coughing fits, muscle pain, reduced oxygen levels, risk of falling and embarrassment when answering the questionnaires. However, to minimize the reported risks, we will carry out the study procedures in a private place to answer the questionnaires and be free not to answer questions that the participant deems embarrassing. The researchers are trained professionals, registered with the regional physiotherapy and occupational therapy council and will be trained and qualified to properly collect data, paying attention to verbal and physical signs of discomfort. Tests and interventions will be carried out in a dry, covered environment, on a non-slip floor, free from personnel movement and adequately signposted. Hand supports during tests and interventions may be used to minimize the risk of falling. The procedures will be carried out with the participant adequately monitored for their vital signs ( heart rate and oxygen level using a pulse oximeter) and will be suspended immediately if any risk to the participant's health is perceived. The participant will be informed that they can suspend the tests at any time they feel uncomfortable, dyspneic, in pain or feel that they are at their limit.

Individuals undergoing research will have a direct health benefit after undergoing 150 minutes of moderate physical activity per week, as recommended by the WHO. In addition to the improvement in clinical characteristics already proven by studies. Potential future benefit through education with classes on the topics: clinical control of the disease, empowerment to develop self-efficacy for physical activity, benefits of exercise, prescription of guided walking with or without a pre-established route, energy conservation techniques for control of symptoms, exercise guidelines to reduce the risk of falling, and motivational strategies in search of active behavior for study participants. And the change in the urban environment, with the development of a more inclusive, safe, sustainable and resilient city.

**6. Schedule**

| ***STEPS/ QUARTERS*** | **01/23** | **02/23** | **03/23** | **04/23** | **08/23** | **09/23** | **11/23** | **02/24** | **10/24** | **12/24** |
| --- | --- | --- | --- | --- | --- | --- | --- | --- | --- | --- |
| Registration and submission of the project to COEP | X | X | X |  |  |  |  |  |  |  |
| Staff training on study procedures | X | X | X |  |  |  |  |  |  |  |
| Participant selection, data collection. |  |  | X | X | X | X | X | X |  |  |
| Database power. |  |  | X | X | X | X | X | X | X | X |
| Analysis and preparation of results for discussion with the team. |  |  |  |  |  |  | X | X |  |  |
| Presentations at partial data scientific events |  |  |  |  | X | X | X |  |  |  |
| Writing scientific articles related to the project. |  |  |  |  |  |  | X | X |  | X |

**7. Budget**

All materials for use in the project are available in the CRDFF master's degree at UFJF, such as accelerometers. The funding materials to be used during the project will be paid for by the researcher himself.

| **Budget Identification** | **Type** | **Value in Reais R$** |
| --- | --- | --- |
| Oximeter Battery | Costing | 700.00 |
| Ink | Costing | 700.00 |
| Paper | Costing | 1500.00 |

**8.References**

1. Kohl HW, 3rd, Craig CL, Lamert EV, Inoue S, Alkandari JR, Leetongin G, et al. The pandemic of physical inactivity: global action for public health. Lancet 2012;380(9838):294-305.

2. Guthold R, et al. Worldwide trends in insufficient physical activity from 2001 to 2016: a pooled analysis of 358 population-based surveys. Lancet GH 2018;6(10):e1077-e1086.

3. Lee IM, et al. Effect of physical inactivity on major non-communicable diseases worldwide: an analysis of burden of disease and life expectancy. Lancet 2012;380(9838):219-229.

4. Ding D, Lawson KD, Kolbe-Alexander TL, et al. The economic burden of physical inactivity: a global analysis of major non-communicable diseases. Lancet 2016;388(10051):1311-1324.

5. Organization WH. Global action plan on physical activity 2018-2030: more active people for a healthier world

6. King AC, Powell KE, Kraus WE. The US Physical Activity Guidelines Advisory Committee Report-Introduction. Med Sci Sports Exerc 2019;51(6):1203-1205.

7. Ministry of Health SdAPàS, Department of Health Promotion. Physical Activity Guide for the Brazilian Population

8. Tudor-Locke C, Craig CL, Brown WJ, Clemes SA, De Cocker K, Giles-Corti B, et al. How many steps/day are enough? For adults. Int J Behav Nutr Phys Act 2011;8:79.

9. Tudor-Locke C, Craig CL, Aoyagi Y, Bell RC, Croteau KA, De Bourdeaudhuij I, et al. How many steps/day are enough? For older adults & special populations. Int J Behav Nutr Phys Act 2011;8:80.

10. Watz H, Waschki B, Meyer T, Magnussen H. Physical activity in patients with COPD. Eur Respir J 2009;33(2):262-272.

11. Pitta F, Troosters T, Spruit MA, et al. Characteristics of physical activities in daily life in chronic obstructive pulmonary disease. Am J Respir Crit Care Med 2005;171(9):972-977.

12. Waschki B, Spruit MA, Watz H, et al. Physical activity monitoring in COPD: compliance and associations with clinical characteristics in a multicenter study. Respir Med 2012;106(4):522-530.

13. Singh S, Morgan MD. Activity monitors can detect brisk walking in patients with chronic obstructive pulmonary disease. J Cardiopulm Rehabil 2001;21(3):143-148.

14. Hernandes NA, Teixeira Dde C, et al. Profile of the level of physical activity in the daily lives of patients with COPD in Brazil. J Bras Pneumol 2009;35(10):949-956.

15. Watz H, Pitta F, Rochester CL, Garcia-Aymerich J, ZuWallack R, Troosters T, et al. An official European Respiratory Society statement on physical activity in COPD. Eur Respir J 2014;44(6):1521-

16. Garcia-Aymerich J, et al. Time-dependent confounding in the study of the effects of regular physical activity in chronic obstructive pulmonary disease.Ann Epidemiol 2008;18(10):775-783.

17. Waschki B, Kirsten A, Holz O, et al. Physical activity is the strongest predictor of all-cause mortality in patients with COPD: a prospective cohort study. Chest 2011;140(2):331-342.

18. Coultas DB, et al. Home-based Physical Activity Coaching, Physical Activity, and Health Care Utilization in Chronic Obstructive Pulmonary Disease. Ann Am Thorac Soc 2018;15(4):470-478.

19. Burge AT, Cox NS, Abramson MJ, Holland AE. Interventions for promoting physical activity in people with chronic obstructive pulmonary disease (COPD). Cochrane Datab. Syst Rev 2020;4

20. Bauman AE, Reis RS, Sallis JF, Wells JC, Loos RJ, Martin BW, et al. Correlates of physical activity: why are some people physically active and others not? Lancet 2012;380(9838):258-271.

21. Sallis JF, Cerin E, Conway TL, et al. Physical activity in relation to urban environments in 14 cities worldwide: a cross-sectional study. Lancet 2016;387(10034):2207-2217.

22. Lo RH. Walkability:what is it? Journal of Urbanism 2009;2:145-166.

23. Bonatto D, Alves FB. Application of Walkability Index for Older Adults' Health in the Brazilian Context: The Case of Vitoria-ES, Brazil. Int J Environ Res Public Health 2022;19(3).

24. Ubiali A, Gori D, Rochira A, Raguzzoni G, Fantini MP. Measures of walkability in the pediatric population: a qualitative review of the literature. Ann Ig 2021;33(1):67-85.

25. Frank LD, Adhikari B, et al. Chronic disease and where you live: Built and natural environmental relationships with physical activity, obesity, and diabetes. Environ Int 2022;158:106959.

26. Murillo R, Reesor-Oyer LM, Hernandez DC, Liu Y, Obasi EM. Neighborhood Walkability and Overweight/Obese Weight Status Among Latino Adults. Am J Health Promot 2020;34(6):599-607.

27. de Courreges A, Occelli F, Muntaner M, et al. The relationship between neighborhood walkability and cardiovascular risk factors in northern France. Sci Total Environ 2021;772:144877.

28. Jones AC, Chaudhary NS, Patki A, et al. Neighborhood Walkability as a Predictor of Incident Hypertension in a National Cohort Study. Front Public Health 2021;9:611895.

29. GOLD. Global Strategy for Prevention, Diagnosis and Management of COPD, 2022.

30. Cox NS, et al. Pulmonary rehabilitation referral and participation are commonly influenced by environment, knowledge, and beliefs about consequences. J Phsysiot 2017; 63:84.

31. Vieira DS, Maltais F, Bourbeau J. Home-based pulmonary rehabilitation in chronic obstructive pulmonary disease patients. Cur Op Pulm Med 2010;16:134-143.

32. Brazilian Society of Pulmonology and Phthisiology. Guidelines for pulmonary function testing. J Bras Pneumol. 2002;28(S3):S44-S58.

33. Pereira CAC, Barreto SP, Simões JG, et al. Reference values for spirometry in a sample of the adult Brazilian population. J Bras Pneumol 1992;18(1):10-22.

34. Da Silv/a G, Morano M, Viana C, Magalhães C, Pereira E. Validation of the COPD assessment test in Portuguese for use in Brazil. J. bras. pneumol. 39 (04).2013.

35. Charlson ME, Szatrowski TP, Peterson J, Gold J. Validation of a combined comorbidity index. J Clin Epidemiol 1994; 47:1245-51

36. Gore, S. et al. Validity and Reliability of Accelerometers in Patients With COPD: A SR. Journal of cardiopulmonary rehabilitation and prevention, 2018: 38, n. 3, p. 147

37. Dillon CB, et al. Number of days required to estimate habitual activity using Wrist-Worn GENEActiv accelerometer: A cross-sectional study. PLoS One2016;11(5):e0109913.

38. Demeyer H, et al. Standardizing the analysis of physical activity in patients with COPD following a pulmonary rehabilitation program. Chest 2014; 46(2):318–327.

39. Rabinovich RA, Louvaris Z, Raste Y, et al. Validity of physical activity monitors during daily life in patients with COPD. Eur Respir J. 2013; 42(5):1205–1215. DOI:10.1183/09031936.00134312

40. Sidhu MS, et al. Patient self-management in primary care patients with mild COPD - protocol of a randomized controlled trial of telephone health coaching. BMC Pulm Med. 2015; 15:16.

41. Bames JB, Benden ME, Biddle S, et al. Letter to the editor: Standardized use of the terms "sedentary" and "sedentary behavior. Appl Ph. Nutr Metab-Physiol . 2012;37:540–542.

42. Kovelis D, et al. Validation of the Modified Pulmonary Functional Status and Dyspnea Questionnaire and the MRC scale for use in Brazilian patients with COPD. JB Pneumol. 2008;34(12):1008.

43. Holland AE, et al. An official European Respiratory Society/American Thoracic Society technical standard: field walking tests in chronic respiratory disease. Eur Respir J. 2014;44(6):1428-46.

44. Borg GA. Psychophysical bases of perceived exertion. Med Sci Sports Exerc 1982;14:377-81.

45. Victor JF, Ximenes LB, Almeida PC. Reliability and validity of the exercise benefits/barriers scale in the elderly. Minutes Paul. sick. 2012;25(1):48-53. https://doi.org/10.1590/S0103- 21002012000800008

46. Brown SA. Measuring perceived benefits and perceived barriers for physical activity. Am J Health Behav. 2005;29(2):107-

47. Sousa TC, Jardim JR, Jones PW. Validation of the Saint George respiratory questionnaire (SGRQ) in chronic obstructive pulmonary disease in Brazil. J Bras Pneumol. 2000;26(3):119-128.

48. Botega NJ, Bio MR, Zomignani MA, Garcia Jr C. Mood disorders in a medical clinic ward and validation of the anxiety and depression measurement scale (HAD). Rev. Public Health 1995; 29(5):359-363.

49. JETTE, AM et al. Late life function and disability instrument: I. Development and evaluation of the disability component. The Journal of Gerontology: Biological Sciences and Medical Sciences, Washington, v. 57, no. 4, p. 209-216, 2002. http://dx.doi.org/10.1093/gerona/57.4.M209. PMid:11909885

50. TOLDRÁ, RC; SOUTO, ACF; BATISTA, MPP; ALMEIDA, MHM Cross-cultural adaptation of the Late-life Function and Disability Instrument into Brazilian Portuguese. Rev. Ter. Ocup. Univ. São Paulo, vol. 23, no. 1, p. 52-61, Jan./Apr. 2012

51. Markland D, Tobin V. A modification to behavioral regulation in exercise questionnaire to include an assessment of amotivation. Journal of Sport and Exercise Psychology. 2004. 26:191-196.

52. Wilson PM, Rodgers WM, Loitz CC & Scime G. “It's who I am…. Really!” The importance of integrated regulation in exercise contexts. Journal of Applied Biobehavioral Research. 2006. 11:79-104.

53. Cid L, Monteiro D, Teixeira D, Teques P, Alves S, Moutão J, Silva M, Palmeira A. The Behavioral Regulation in Exercise Questionnaire (BREQ-3) Portuguese-Version: Evidence of Reliability, Validity and Invariance Across Gender . Front Psychol. 2018 Oct 11;9:1940. doi: 10.3389/fpsyg.2018.01940.

54. Florindo, Alex Antonio et al. Validation of an environmental perception scale for the practice of physical activity in adults from a region of low socioeconomic status. Brazilian Journal of Kinanthropometry & Human Performance [online]. 2012, vol. 14, no. 6 [Accessed 3 January 2023], pp. 647-659.

55. Saelens BE, Sallis JF, Black JB, Chen D. Neighborhood-based differences in physical activity: an environmental scale evaluation. Am J Public Health 2003;93:1552-8.

56. Malavasi L, Duarte M, Both J, Reis R. Active mobility scale in the community environment - NEWS Brazil: retranslation and reproducibility. Rev Bras Cineantropom Performance Hum 2007;9:339-50.

57. Reis MS, Reis RS, Hallal PC. Validity and reliability of a physical activity social support assessment scale. Rev Saude Publica 2011;45(2):294-301
