# Supplementary material for: Exploring the impact of the environment on physical activity in patients with chronic obstructive pulmonary disease (EPCOT)—A comparative analysis between suggested and free walking: Protocol study
Source: PLoS One. 2024 Aug 13;19(8):e0306045. doi: 10.1371/journal.pone.0306045 (PMC11321554; doi:10.1371/journal.pone.0306045)
Supplement: S2 Protocol — (DOCX) [file pone.0306045.s006.docx]

**HOSPITAL UNIVERSITÁRIO DA UNIVERSIDADE FEDERAL DE JUIZ DE FORA**

## Comitê de Ética em Pesquisa em Seres Humanos do HU-UFJF

## *Comitê de Ética em Pesquisa HU - UFJF*

Dados do Projeto e do (a) Coordenador do Projeto

| **Título do Projeto** | *Determinantes ecológicos do comportamento ativo de pessoas com Doença Pulmonar Obstrutiva Crônica e efeitos da caminhabilidade* |
| --- | --- |
| **Pesquisadores** | Larissa Guimarães Paiva  Nara Batista de Souza  Túlio Medina Dutra de Oliveira  Anderson José  Carla Malaguti |
| **Contato:** |  |
| **Unidade/Departamento:** | Faculdade de Fisioterapia / Departamento Fisioterapia Cardiorrespiratória e Musculoesquelética |
| **Setor/Unidade onde a pesquisa será realizada no**  **HU-UFJF/Ebserh (quando aplicável):** | Unidade Multiprofissional/ Ambulatório de Reabilitação Cardiopulmonar |
| **Data:** | 04/01/2023 |

***1.*Justificativa/Caracterização do Problema**

A inatividade física é uma característica comum de muitas doenças crônicas, tanto como causa quanto como uma consequência. A alta prevalência de inatividade física é um problema^1,2^ que contribui para aumentar a morbidade, taxas mais altas de morte prematura^3^, e aumento dos custos^4^. A Organização Mundial de Saúde (OMS)^5^, o American College Sports of Medicine^6^, assim como o Guia de Atividade Física para a População Brasileira^7^ recomendam acumular 150 minutos semanais de atividade física moderada, ou 75 minutos de atividade física vigorosa, o que traduz em benefícios relacionados à saúde. Ainda, recomenda-se também o acúmulo de 7.500 a 10.000 passos por dia para adultos ^8^, e cerca de 5.000 passos por dia para idosos e populações especiais^9^, como nas doenças pulmonares crônicas. Em pacientes com doença pulmonar obstrutiva crônica (DPOC) foram observados níveis signiﬁcantemente mais baixos de atividade física em comparação com controles saudáveis ^10-12^. Os dados existentes mostram que o tempo gasto andando é menor em pacientes com DPOC em comparação com pessoas saudáveis da mesma idade^11,13,14^. Também têm sido observadas associações entre atividade física e características clínicas dos pacientes com DPOC, como gravidade da doença, comorbidades, exacerbações e fatores comportamentais^15^. Estudos longitudinais prospectivos mostraram associação de baixos níveis de atividade física com aumento do número de exacerbações, risco de internações e morte por todas as causas em pacientes com DPOC^16-17^. Estudos têm focado em testar a eﬁcácia de intervenções como programas de reabilitação pulmonar, tratamento farmacológico, oxigênio, além de intervenções comportamentais para aumentar a atividade física em pacientes com DPOC^18-19^. Porém, essas intervenções têm focado apenas no nível de fatores individuais, e não têm resultado em modiﬁcações duradouras de estilo de vida mais ativo dessa população^19^. Por outro lado, deve-se reconhecer que, em geral, a atividade física depende de muitos fatores, além dos biológicos, comportamentais e genéticos, como também fatores sociais, ambientais, culturais e de políticas públicas. O modelo ecológico tem uma visão ampla da causa do comportamento inativo, com o fator social e o meio ambiente também incluídos como contribuintes, particularmente externos ao fator saúde, como as áreas de planejamento urbano, sistemas de transporte e espaços públicos abertos como praças, parques e ciclovias ^20^. O ambiente urbano tem o potencial de contribuir substancialmente para a atividade física. Já foi demonstrado que viver em cidades amigáveis para a atividade física podem ajudar o morador a atingir cerca de 45-59% dos 150min/semana das diretrizes recomendadas para a atividade física^21^. A caminhabilidade refere-se ao quanto uma área é propícia para caminhadas e transporte ativo ^22^. A caminhabilidade tem sido objeto de investigação crescente na área da saúde, incluindo diferentes populações como idosos^23^, crianças e adolescentes^24^, e condições como risco de diabetes^25^, obesidade^25,26^ e risco cardiovascular^27,28^. Contudo, o modelo ecológico envolvendo o fator ambiental como determinante da atividade física na DPOC, ainda não tem sido investigado. Como a atividade física é afetada por diversos fatores, o modelo ecológico (incluindo interrelações entre fatores pessoais e seus ambientes físicos) abrangente parece explicar a atividade física, propondo que determinantes em todos os níveis - individual, social, ambiental e político - são contribuintes. Nesse sentido, esse projeto visa investigar desde os fatores individuais (idade, sexo, capacidade física, estado de saúde, comorbidades, motivação e percepção), fatores interpessoais (como participação social e comunitária) e fatores ambientais (como a caminhabilidade), que possam inﬂuenciar a atividade física de pessoas com DPOC e que deve ser considerada para trazer subsídios para traçar estratégias de melhor adesão para atividade física dessa população. Secundariamente, um ensaio clínico controlado comparando grupo de caminhada orientada em rotas de melhor caminhabilidade com grupo de caminhada em percurso livre, poderá fornecer subsídios do impacto ambiental na adesão à prática de atividade física dessa população.

**2.Objetivos**

**2.1 Geral**

O objetivo desse estudo investigar fatores individuais (idade, sexo, capacidade física, estado de saúde, comorbidades, motivação, ansiedade e depressão, qualidade de vida e percepção), interpessoais (como participação social e comunitária) e fatores ambientais (como a caminhabilidade) que possam inﬂuenciar a atividade física de pessoas com DPOC. Ao identiﬁcar rotas de melhor caminhabilidade, realizar um ensaio clínico controlado comparando um grupo de caminhada orientada em rotas pré-estabelecidas de melhor caminhabilidade com um grupo de caminhada em percurso livre.

**2.2 Específicos**

1: Avaliar os indicadores individuais: idade, sexo, gravidade da doença, capacidade física, estado de saúde, comorbidades, motivação, auto-eﬁcácia para o exercício, níveis de ansiedade e depressão, qualidade de vida, percepção de barreiras e benefícios para atividade física e nível de atividade física medida objetivamente através de acelerômetro; avaliar os indicadores interpessoais por meio da participação social e na comunidade; avaliar os indicadores ambientais por meio da caminhabilidade na comunidade dos participantes. 2: Identiﬁcar possíveis relações e fatores determinantes para a atividade física dos pacientes com DPOC. 3: Realizar intervenção educacional potencializando as facilidades e a dissolução de barreiras, envolvendo temas como controle clínico da doença, empoderamento para desenvolver a autoeﬁcácia para a atividade física, benefícios do exercício, prescrição de caminhada orientada com ou sem percurso pré-estabelecido, técnicas de conservação de energia para controle dos sintomas, orientações de exercícios para redução do risco de queda, e estratégias motivacionais em busca de um comportamento ativo para os participantes do estudo. 4: Avaliar a efetividade da prática de caminhada orientada em rotas ou trilhas de melhor caminhabilidade comparada a caminhada em percurso livre, na adesão a prática da atividade física na comunidade, bem como seus efeitos nos indicadores pessoais e interpessoais. 5:Elaborar relatórios para a secretaria de saúde e de planejamento urbano da cidade de Juiz de Fora, bem como para as mídias locais, a ﬁm de sensibilizar para o desenvolvimento de ações para alcançar uma cidade amigável, com mais áreas de caminhabilidade, favorável a prática de atividade física segura a todos.

**3.Metodologia e Estratégias de Ação**

**Desenho do Estudo**

Trata-se de um estudo prospectivo a ser conduzido a partir da data de aprovação do Comitê de Ética em Pesquisa do hospital Universitário da Universidade Federal de Juiz de Fora. Todos os pacientes que concordarem em participar do estudo assinarão o Termo de Consentimento Livre e Esclarecido (TCLE). Os indivíduos serão recrutados a partir da lista de pacientes com diagnóstico de DPOC encaminhados para o serviço de fisioterapia e reabilitação pulmonar do Hospital Universitário da Universidade Federal de Juiz de Fora e Unidades de Atenção Primária a saúde do município de Juiz de Fora. Todos passarão por uma entrevista e avaliação de fatores multiníveis para a prática de atividade física dos pacientes com DPOC para obter seus determinantes (Etapa 1), e com posterior convite para o ensaio clínico controlado (Etapa 2) com orientações para atividade física por meio de caminhadas com rotas guiadas de melhor caminhabilidade (grupo experimental) comparada a orientações de caminhada em percurso livre (grupo controle).

**Participantes**

Serão estudados indivíduos com diagnóstico confirmado de DPOC baseado nos critérios da *Global Initiative for Chronic Obstructive Lung Disease* (GOLD), com a relação entre o volume expiratório forçado pós-broncodilatador no primeiro segundo (VEF_1_)/capacidade vital forçada (CVF) sendo <70% e VEF_1_ < 80% do valor predito^29^, de ambos os sexos, com idade maior de 18 anos, estáveis clinicamente, sem doenças cardiovasculares instáveis, distúrbios neurológicos, osteomusculares e em condições de realizar as avaliações e intervenção propostas. Serão excluídos do estudo os participantes incapazes de realizar ou compreender as avaliações do estudo devido a deficiência física ou psicológica e um diagnóstico primário de uma doença respiratória diferente da DPOC, em suporte de oxigenoterapia, com limitações ortopédicas e/ou neurológicas que impeçam a realização dos testes, hospitalização recente (últimos 3 meses), comprometimento cognitivo identificado pela pontuação menor ou igual a 4 no *Six Item Screener* ^30,31^, ou que expressaram seu desejo de se retirar do estudo.

**Procedimentos e medidas:**

Na Etapa 1, serão obtidos dados individuais como informações clínico-demográﬁcas, de gravidade da doença, comorbidades, de capacidade de exercício, nível de atividade física, atividades de vida diária, ansiedade e depressão, risco e medo de sofrer queda, qualidade de vida, motivação e autoeﬁcácia para o exercício, além de barreiras e benefícios para a atividade física. Também serão obtidas informações interpessoais por meio de instrumentos de participação social, de participação na comunidade; além de informações de nível ambiental como medidas de caminhabilidade. Estas avaliações serão realizadas em duas visitas para não consumir tempo e cansaço do participante. Na Etapa 2, após as avaliações e a análise para determinação dos fatores que impactam a atividade física dessa população, os pacientes serão alocados de forma aleatória em grupos de caminhada orientada em percurso de melhor caminhabilidade ou em grupo de caminhada orientada sem sugestão de percurso. O período de intervenção se dará por 8 semanas, com sugestões de caminhadas de 3-5 vezes por semana e com fornecimento de diário para registro das caminhadas. Todos os participantes receberão intervenções educacionais por meio de aulas expositivas e material impresso. A supervisão indireta de ambos os grupos se dará por meio de chamadas telefônicas ou mensagens de texto quinzenalmente. Após a conclusão do período de intervenção, a reavaliação será realizada. A função cognitiva global será avaliada através do *Six-item Screener* que avalia a lembrança de 3 itens e orientação para ano, mês e dia da semana, produzindo uma pontuação de 0 a 6 questões respondidas corretamente. Uma pontuação ≤ 4 é considerada positiva para comprometimento cognitivo^30,31^ e indicará exclusão do participante no protocolo.

**Variáveis de caracterização da amostra:**

**Função Pulmonar**

A espirometria será realizada por meio de um espirômetro portátil (Spirobank II, Medical International Research, New Berlin, USA). Os procedimentos técnicos, os critérios de aceitabilidade e reprodutibilidade adotados serão determinados de acordo com as Diretrizes para testes de função pulmonar ^32^. A manobra será explicada e demonstrada na prática pelos pesquisadores previamente treinados. Será solicitado ao participante realizar três manobras reprodutíveis de expiração máxima forçada, sustentando a expiração durante o período de presença do fluxo expiratório.  Os dados serão expressos em valores absolutos e porcentagem do previsto para população brasileira ^33^.

**Impacto dos Sintomas**

O impacto dos sintomas da DPOC será avaliado pelo Questionário *COPD Assessment Test* (CAT), instrumento para a quantificação do impacto dos sintomas da DPOC na vida diária. O CAT é composto de oito itens, denominados tosse, expectoração, aperto no peito, falta de ar, limitações nas atividades domiciliares, confiança em sair de casa, sono e energia. Para cada item, o paciente escolhe apenas uma opção de resposta, cuja pontuação varia de zero a cinco. Os resultados variam de acordo com a faixa dos escores obtidos, classificados em relação ao impacto clínico: 6-10 pontos: leve; 11-20: moderado; 21-30: grave; 31-40: muito grave ^34^.

**Comorbidades**

O índice de Charlson será utilizado para avaliar a presença de comorbidades. Esse instrumento vem sendo amplamente usado em pesquisas para identificação de condições médicas que não se encontram em prontuários. No contexto da prática clínica, o ICC auxilia os profissionais a classificar pacientes baseando-se na gravidade das doenças, criando esquemas adequados de alocação de recursos. O índice é composto por 19 condições clínicas, selecionadas com base no efeito sobre o prognóstico do paciente em termos de mortalidade. Para cada uma das condições clínicas uma pontuação é estabelecida com base no risco relativo e com pesos variando de 0 a 6 ^35^.

**Desfecho Primário:**

O desfecho primário será dará através avaliação objetiva do nível de atividade física, que será avaliada pelo acelerômetro Actigraph.

**Nível de Atividade Física**

Será realizada uma avaliação objetiva do nível de atividade física do participante por meio do acelerômetro Actigraph GT3X® (Actigraph LLC, USA), monitor de atividade física validado e confiável para uso em indivíduos com DPOC ^36^. Os participantes usarão uma cinta elástica que fixa o dispositivo no nível da cintura do membro inferior dominante, sendo orientados a retirá-lo apenas ao tomar banho, realizar atividades aquáticas e durante o sono. O dispositivo tem tamanho e dimensões mínimas que não afetam o conforto durante sua utilização, será ofertado a cada participante junto a um manual contendo as informações e instruções sobre o uso do dispositivo, e um diário para preenchimento sobre o dia da semana e os horários de fixação e de retirada do dispositivo. Para que os dados de atividade física sejam válidos para análise, os participantes precisam usar o acelerômetro por pelo menos 4 dias, abrangendo pelo menos um dia de fim de semana^37,38^. Um dia de dados será incluído se houver pelo menos 10 horas de uso em um período de 24 horas (0:00-23:59 horas)^39^. Apenas os períodos de vigília serão retidos na análise. A conversão padrão de 1 MET = 3,5 mL /kg/min será usada e então codificada em uma das quatro categorias de intensidade absoluta: sedentário (<1,5 METs), leve (1,5–2,99 METs), moderado (3,00–5,99 METs), ou atividade vigorosa (> 6 METs). A AF média diária será calculada como o tempo total de atividade física (de todas as intensidades de atividade física) dividido pelo número de dias elegíveis. O tempo de atividade física também será estratificado de acordo com a intensidade (ou seja, intensidade leve e intensidade moderada-vigorosa) e comportamento sedentário para análise^40,41^.

**Desfecho Secundários:**

**Dispneia**

Os pacientes irão relatar sua dispneia de acordo com a escala Medical Research Council modificada ^42^, previamente traduzido, adaptado culturalmente e validado para a população brasileira. Esta escala é composta por cinco atividades cuja graduação de dispneia varia de 0 a 4. Quanto maior o escore maior o sintoma de dispnéia relatado.

**Capacidade de exercício**

A capacidade de exercício será avaliada pelo Teste de Caminhada de Seis Minutos (TC6) é considerado um teste submáximo usado como uma medida única do estado funcional. Será realizado segundo as orientações das diretrizes internacionais para a aplicação do teste^43^, em um corredor com 30 metros de comprimento e com superfície lisa, onde os participantes serão instruídos a caminhar a maior distância possível durante os seis minutos. A cada minuto, o examinador informará aos participantes o tempo que falta para completar os seis minutos e emitirá frases de incentivo padronizadas.^43^ Será permitido ao participante descansar caso este necessite, porém o cronômetro não será interrompido. Serão realizados dois testes com um intervalo de 30 minutos de repouso entre eles. O teste com a maior distância percorrida será considerado para análise. A frequência cardíaca e a saturação da hemoglobina de pulso (SpO_2_) serão continuamente monitorizadas. Serão mensuradas também a pressão arterial sistêmica, os sintomas de dispneia e a fadiga pela escala de Borg modificada ^44^ antes e após o teste. O teste poderá ser interrompido pelo próprio participante ou pelo avaliador caso haja mal-estar, náuseas, dispneia importante, fadiga extrema, precordialgia, cefaleia ou se SpO_2_<85%.

**Barreiras percebida para atividade física**

Os benefícios percebidos e as barreiras à atividade física serão avaliados com o EBBS Brasil, um questionário de 42 itens: 14 pertencentes à Barrier Scale (EBBSBAR) e 28 à Benefits Scale (EBBSBEN). O escore EBBSBEN foi calculado pela soma de 28 itens em cinco domínios: aspectos biológicos, desempenho físico, aspectos psicológicos, interação social e saúde preventiva. O escore EBBSBAR foi calculado pela soma de 14 itens em quatro domínios: gasto de tempo, esforço físico, ambiente de exercício e desânimo familiar. Valores mais altos indicaram maiores benefícios ou barreiras percebidas ^45,46^.

**Qualidade de Vida Relacionada à Saúde**

A qualidade de vida será avaliada pelo questionário específico para doenças respiratórias Saint

George’s Respiratory Questionnaire (SGRQ), que já foi traduzido, adaptado culturalmente e validado para a população brasileira^47^. O instrumento aborda os aspectos relacionados a três domínios:

sintomas, atividade e impactos psicossociais que a doença respiratória inflige ao paciente. Cada domínio tem uma pontuação máxima possível e sua pontuação total e para cada domínio varia de 0 a 100, quanto menor o valor, melhor a qualidade de vida.

**Ansiedade e Depressão**

A ansiedade e depressão será mensurada por meio da Hospital Anxiety and Depression Scale (HADS). Esta escala foi desenvolvida para estimar a prevalência de ansiedade e de depressão em adultos e atualmente é utilizada também em pacientes com doenças pulmonares crônicas.^48^ A escala tem o objetivo de identificar casos (possíveis ou prováveis) de transtornos de ansiedade e/ou depressão. É composta por 14 itens divididos em duas subescalas: sete questões para o diagnóstico do Transtorno de Ansiedade e outras sete para o Transtorno Depressivo. A escala de resposta varia entre zero a três pontos (de ausente a muito frequente) com escore máximo de 21 pontos por subescala. As pontuações mais altas indicam maior gravidade da ansiedade e da depressão^48^.

**Participação Social**

Será utilizado o questionário Late-Life Function and Disability Instrument (LLFDI)^49,50^ é um instrumento abrangente e sensível desenvolvido para a documentação de mudanças consequentes ao processo de envelhecimento, permite capturar o desempenho funcional da pessoa nos ambientes doméstico e comunitário. O instrumento apresenta dois componentes: Incapacidade e Função, que constituem escalas distintas. Será utilizado apenas o primeiro, Incapacidade, que documenta a frequência e a limitação do indivíduo para realização de 16 atividades de vida diária, compreendendo as atividades básicas, instrumentais e avançadas. Além dos escores totais (Frequência total e Limitação total), é possível obter um escore para cada domínio que compõe essa escala, a saber, Papel pessoal, Papel social, Papel instrumental e Papel de gerenciamento.

**Motivação para Exercício**

O Behavioral Regulation Exercise Questionnaire (BREQ) será usado para avaliar padrões de comportamento autodeterminado na prática do exercício físico. Foi criado em 1997^59^ e adaptado em outras duas versões. A versão mais atual é o BREQ-3^51,52^, é um instrumento traduzido e adaptado para a língua portuguesa^53^, tem 23 itens precedidos pelo enunciado “Porque você pratica exercício físico?” respondidos numa escala tipo Likert de 5 pontos, em que o respondente indica o grau de concordância que mais se ajusta ao seu caso que varia entre “não é verdade para mim” (0) até “muitas vezes é verdade para mim”(4). Estes 23 itens organizam-se 6 domínios: a motivação, regulação externa, introjetada, identificadas, integradas (motivação extrínseca) e regulação intrínseca (motivação intrínseca), as seis subescalas que compõem o BREQ-3 permite a análise do perfil de motivação para a prática de exercício físico mediante o chamado Índice de Autodeterminação (IaD) onde, diferentes pesos são atribuídos a cada subescala, recebendo as subescalas autônomas pesos positivos e as subescalas menos autodeterminada, pesos negativos.

**Caminhabilidade**

A caminhabilidade será coletada usando a escala de percepção do ambiente para a prática de atividade física ^54^, composta por questões embasadas na escala NEWS ^55,56^ e numa escala de apoio social para a prática de atividade física ^57^. A versão e composta de 38 questões foi validada para adultos brasileiros^54^. Esta ferramenta inclui características do ambiente (construído, natural e social): acesso ao comércio e locais de prática de AF, segurança no trânsito, segurança em relação a crimes, estética e satisfação do bairro, qualidade de ruas e calçadas, iluminação, poluição e suporte social para a atividade física.

**Análise dos dados**

Os dados serão analisados no programa SPSS v.22.0.

**4. Resultados e os impactos esperados**

Espera-se com o desenvolvimento desta proposta sejam identiﬁcados os indicadores multiníveis, para assim potencializar as facilidades e dissolver as barreiras por meio de intervenção educacional e de atividade física por meio da caminhada orientada, voltadas para a promoção da atividade física de pessoas com DPOC, traduzindo em uma população clínica mais ativa. Produzir relatórios com os indicadores urbanísticos que impactam na promoção da atividade física e saúde e prevenção de agravos para as secretarias de saúde e de planejamento urbano do município, bem como conteúdo para divulgação nas mídias locais.

**5. Riscos e benefícios**

Os riscos e desconfortos oferecidos serão aqueles decorrentes dos testes, consistem em cansaço muscular, dispneia, crise de tosse, dor muscular, redução dos níveis de oxigênio, risco de queda e constrangimento ao responder os questionários. Entretanto, para minimizar os riscos relatados realizaremos os procedimentos do estudo em local reservado para responder aos questionários e liberdade para não responder questões que o participante julgar constrangedoras. Os pesquisadores são profissionais formados, com registro no conselho regional de fisioterapia e terapia ocupacional e estarão treinados e habilitados para a adequada coleta de dados, atentos aos sinais verbais e físicos de desconforto. Os testes e intervenções serão realizados em ambiente seco, coberto, em piso não derrapante, livre de movimentação de pessoal e adequadamente sinalizados. Apoios de mão durante os testes e intervenções poderão ser utilizados para minimizar os riscos de queda. Os procedimentos serão realizados com o participante adequadamente monitorizado em seus sinais vitais (frequência cardíaca e nível de oxigênio por meio do oxímetro de pulso) e serão suspensos imediatamente ao se perceber algum risco à saúde do participante. Será informado ao participante que ele poderá suspender os testes a qualquer momento em que se sentir desconfortável, dispneico, dor ou sentir que está em seu limite.

Os indivíduos submetidos a pesquisa terão benefício direto na saúde após serem submetidos a 150 minutos de atividade física moderada por semana, assim como recomendado pela OMS. Além da melhora de características clínicas já comprovada por estudos. Benefício potencial futuro através da educação com aulas com os temas: controle clínico da doença, empoderamento para desenvolver a autoeﬁcácia para a atividade física, benefícios do exercício, prescrição de caminhada orientada com ou sem percurso pré-estabelecido, técnicas de conservação de energia para controle dos sintomas, orientações de exercícios para redução do risco de queda, e estratégias motivacionais em busca de um comportamento ativo para os participantes do estudo. E a mudança no ambiente urbano, com desenvolvimento de uma cidade mais inclusiva, segura, sustentável e resiliente.

**6. Cronograma**

| ***ETAPAS/ TRIMESTRES*** | **01/23** | **02/23** | **03/23** | **04/23** | **08/23** | **09/23** | **11/23** | **02/24** | **10/24** | **12/24** |
| --- | --- | --- | --- | --- | --- | --- | --- | --- | --- | --- |
| Registro e envio do projeto ao COEP | X | X | X |  |  |  |  |  |  |  |
| Treinamento da equipe aos procedimentos do estudo | X | X | X |  |  |  |  |  |  |  |
| Seleção de participantes, coleta de dados. |  |  | X | X | X | X | X | X |  |  |
| Alimentação da base de dados. |  |  | X | X | X | X | X | X | X | X |
| Análises e preparação de resultados para discussão com equipe. |  |  |  |  |  |  | X | X |  |  |
| Apresentações em eventos científicos de dados parciais |  |  |  |  | X | X | X |  |  |  |
| Redação de artigos científicos relacionados ao projeto. |  |  |  |  |  |  | X | X |  | X |

**7.Orçamento**

Todos os materiais para uso no projeto estão disponíveis no mestrado CRDFF da UFJF como os acelerômetros. Os materiais de custeio a serem utilizados durante o projeto serão custeados pelo próprio pesquisador.

| **Identificação do Orçamento** | **Tipo** | **Valor em Reais R$** |
| --- | --- | --- |
| Bateria Oximetro | Custeio | 700,00 |
| Tinta | Custeio | 700,00 |
| Papel | Custeio | 1500,00 |

**8.Referências**

1. Kohl HW, 3rd, Craig CL, Lamert EV, Inoue S, Alkandari JR, Leetongin G, et al. The pandemic of physical inactivity: global action for public health. Lancet 2012;380(9838):294-305.

2. Guthold R, et al. Worldwide trends in insufficient physical activity from 2001 to 2016: a pooled analysis of 358 population-based surveys. Lancet GH 2018;6(10):e1077-e1086.

3. Lee IM, et al. Effect of physical inactivity on major non-communicable diseases worldwide: an analysis of burden of disease and life expectancy. Lancet 2012;380(9838):219-229.

4. Ding D, Lawson KD, Kolbe-Alexander TL, et al. The economic burden of physical inactivity: a global analysis of major non-communicable diseases. Lancet 2016;388(10051):1311-1324.

5. Organization WH. Global action plan on physical activity 2018-2030: more active people for a healthier world

6. King AC, Powell KE, Kraus WE. The US Physical Activity Guidelines Advisory Committee Report-Introduction. Med Sci Sports Exerc 2019;51(6):1203-1205.

7. Ministério da Saúde SdAPàS, Departamento de Promoção da Saúde. Guia de Atividade Física para a População Brasileira

8. Tudor-Locke C, Craig CL, Brown WJ, Clemes SA, De Cocker K, Giles-Corti B, et al. How many steps/day are enough? For adults. Int J Behav Nutr Phys Act 2011;8:79.

9. Tudor-Locke C, Craig CL, Aoyagi Y, Bell RC, Croteau KA, De Bourdeaudhuij I, et al. How many steps/day are enough? For older adults & special populations. Int J Behav Nutr Phys Act 2011;8:80.

10. Watz H, Waschki B, Meyer T, Magnussen H. Physical activity in patients with COPD. Eur Respir J 2009;33(2):262-272.

11. Pitta F, Troosters T, Spruit MA, et al. Characteristics of physical activities in daily life in chronic obstructive pulmonary disease. Am J Respir Crit Care Med 2005;171(9):972-977.

12. Waschki B, Spruit MA, Watz H, et al. Physical activity monitoring in COPD: compliance and associations with clinical characteristics in a multicenter study. Respir Med 2012;106(4):522-530.

13. Singh S, Morgan MD. Activity monitors can detect brisk walking in patients with chronic obstructive pulmonary disease. J Cardiopulm Rehabil 2001;21(3):143-148.

14. Hernandes NA, Teixeira Dde C, et al. Profile of the level of physical activity in the daily lives of patients with COPD in Brazil. J Bras Pneumol 2009;35(10):949-956.

15. Watz H, Pitta F, Rochester CL, Garcia-Aymerich J, ZuWallack R, Troosters T, et al. An official European Respiratory Society statement on physical activity in COPD. Eur Respir J 2014;44(6):1521-

16. Garcia-Aymerich J, et al. Time-dependent confounding in the study of the effects of regular physical activity in chronic obstructive pulmonary disease.Ann Epidemiol 2008;18(10):775-783.

17. Waschki B, Kirsten A, Holz O, et al. Physical activity is the strongest predictor of all-cause mortality in patients with COPD: a prospective cohort study. Chest 2011;140(2):331-342.

18. Coultas DB, et al. Home-based Physical Activity Coaching, Physical Activity, and Health Care Utilization in Chronic Obstructive Pulmonary Disease. Ann Am Thorac Soc 2018;15(4):470-478.

19. Burge AT, Cox NS, Abramson MJ, Holland AE. Interventions for promoting physical activity in people with chronic obstructive pulmonary disease (COPD). Cochrane Datab. Syst Rev 2020;4

20. Bauman AE, Reis RS, Sallis JF, Wells JC, Loos RJ, Martin BW, et al. Correlates of physical activity: why are some people physically active and others not? Lancet 2012;380(9838):258-271.

21. Sallis JF, Cerin E, Conway TL, et al. Physical activity in relation to urban environments in 14 cities worldwide: a cross-sectional study. Lancet 2016;387(10034):2207-2217.

22. Lo RH. Walkability:what isit? Journal of Urbanism 2009;2:145-166.

23. Bonatto D, Alves FB. Application of Walkability Index for Older Adults' Health in the Brazilian Context: The Case of Vitoria-ES, Brazil. Int J Environ Res Public Health 2022;19(3).

24. Ubiali A, Gori D, Rochira A, Raguzzoni G, Fantini MP. Measures of walkability in the pediatric population: a qualitative review of the literature. Ann Ig 2021;33(1):67-85.

25. Frank LD, Adhikari B, et al. Chronic disease and where you live: Built and natural environment relationships with physical activity, obesity, and diabetes. Environ Int 2022;158:106959.

26. Murillo R, Reesor-Oyer LM, Hernandez DC, Liu Y, Obasi EM. Neighborhood Walkability and Overweight/Obese Weight Status Among Latino Adults. Am J Health Promot 2020;34(6):599-607.

27. de Courreges A, Occelli F, Muntaner M, et al. The relationship between neighbourhood walkability and cardiovascular risk factors in northern France. Sci Total Environ 2021;772:144877.

28. Jones AC, Chaudhary NS, Patki A, et al. Neighborhood Walkability as a Predictor of Incident Hypertension in a National Cohort Study. Front Public Health 2021;9:611895.

29. GOLD. Global Strategy for Prevention, Diagnosis and Management of COPD, 2022.

30. Cox NS, et al. Pulmonary rehabilitation referral and participation are commonly influenced by environment, knowledge, and beliefs about consequences. J Phsysiot 2017; 63:84.

31. Vieira DS, Maltais F, Bourbeau J. Home-based pulmonary rehabilitation in chronic obstructive pulmonary disease patients. Cur Op Pulm Med 2010;16:134-143.

32. Sociedade Brasileira de Pneumologia e Tisiologia. Diretrizes para testes de função pulmonar. J Bras Pneumol. 2002;28(S3):S44-S58.

33. Pereira CAC, Barreto SP, Simões JG, et al. Valores de referência para espirometria em uma amostra da população brasileira adulta. J Bras Pneumol 1992;18(1):10-22.

34. Da Silv/a G, Morano M, Viana C, Magalhães C, Pereira E. Validação do teste de avaliação da DPOC em português para uso no Brasil. J. bras. pneumol. 39 (04).2013.

35. Charlson ME, Szatrowski TP, Peterson J, Gold J. Validation of a combined comorbidity index. J Clin Epidemiol 1994; 47:1245-51

36. Gore, S. et al. Validity and Reliability of Accelerometers in Patients With COPD: A SR. Journal of cardiopulmonary rehabilitation and prevention, 2018: 38, n. 3, p. 147

37. Dillon CB, et al. Number of days required to estimate habitual activity using Wrist-Worn GENEActiv accelerometer: A cross-sectional study. PLoS One2016;11(5):e0109913.

38. Demeyer H, et al. Standardizing the analysis of physical activity in patients with COPD following a pulmonary rehabilitation program. Chest 2014; 46(2):318–327.

39. Rabinovich RA, Louvaris Z, Raste Y, et al. Validity of physical activity monitors during daily life in patients with COPD. Eur Respir J. 2013; 42(5):1205–1215. DOI:10.1183/09031936.00134312

40. Sidhu MS, et al. Patient self-management in primary care patients with mild COPD - protocol of a ran-domised controlled trial of telephone health coaching. BMC Pulm Med. 2015; 15:16.

41. Bames JB, Benden ME, Biddle S, et al. Letter to the editor: Standardized use of the terms "sedentary" and "sedentary be-haviours. Appl Ph. Nutr Metab-Physiol . 2012;37:540–542.

42. Kovelis D, et al. Validation of the Modified Pulmonary Functional Status and Dyspnea Questionnaire and the MRC scale for use in Brazilian patients with COPD. J B Pneumol. 2008;34(12):1008.

43. Holland AE, et al. An official European Respiratory Society/American Thoracic Society technical standard: field walking tests in chronic respiratory disease. Eur Respir J. 2014;44(6):1428-46.

44. Borg GA. Psychophysical bases of perceived exertion. Med Sci Sports Exerc 1982;14:377-81.

45. Victor JF, Ximenes LB, Almeida PC. Reliability and validity of the exercise benefits/barriers scale in the elderly. Acta paul. enferm. 2012;25(1):48-53. https://doi.org/10.1590/S0103- 21002012000800008

46. Brown SA. Measuring perceived benefits and perceived barriers for physical activity. Am J Health Behav. 2005;29(2):107-

47. Sousa TC, Jardim JR, Jones PW. Validation of the Saint George respiratory questionnaire (SGRQ) in chronic obstructive pulmonary disease in Brazil. J Bras Pneumol. 2000;26(3):119-128.

48. Botega NJ, Bio MR, Zomignani MA, Garcia Jr C. Transtornos do humor em enfermaria de clínica médica e validação de escala de medida (HAD) de ansiedade e depressão. Rev. Saúde  Pública 1995; 29(5):359-363.

49. JETTE, A. M. et al. Late life function and disability instrument: I. Development and evaluation of the dis- ability component. The Journal of Gerontology: Biologi- cal Sciences and Medical Sciences, Washington, v. 57, n. 4, p. 209-216, 2002. http://dx.doi.org/10.1093/ gerona/57.4.M209. PMid:11909885

50. TOLDRÁ, R. C.; SOUTO, A. C. F.; BATISTA, M. P. P.; ALMEIDA, M. H. M. Adaptação transcultural do Late-life Function and Disability Instrument para o Português Brasileiro. Rev. Ter. Ocup. Univ. São Paulo, v. 23, n. 1, p. 52-61, jan./abr. 2012

51. Markland D, Tobin V. A modification to behavioural regulation in exercise questionnaire to include an assessment of amotivation. Journal of Sport and Exercise Psychology. 2004. 26:191-196.

52. Wilson PM, Rodgers WM, Loitz CC & Scime G. “It’s who I am …. Really!”. The importance of integrated regulation in exercise contexts. Journal of Applied Biobehavioral Research. 2006. 11:79-104.

53. Cid L, Monteiro D, Teixeira D, Teques P, Alves S, Moutão J, Silva M, Palmeira A. The Behavioral Regulation in Exercise Questionnaire (BREQ-3) Portuguese-Version: Evidence of Reliability, Validity and Invariance Across Gender. Front Psychol. 2018 Oct 11;9:1940. doi: 10.3389/fpsyg.2018.01940.

54. Florindo, Alex Antonio et al. Validação de uma escala de percepção do ambiente para a prática de atividade física em adultos de uma região de baixo nível socioeconômico. Revista Brasileira de Cineantropometria & Desempenho Humano [online]. 2012, v. 14, n. 6 [Acessado 3 Janeiro 2023], pp. 647-659.

55. Saelens BE, Sallis JF, Black JB, Chen D. Neighborhood-based differences in physical activity: an environment scale evaluation. Am J Public Health 2003;93:1552-8.

56. Malavasi L, Duarte M, Both J, Reis R. Escala de mobilidade ativa no ambiente comunitário - NEWS Brasil: retraducao e reprodutibilidade. Rev Bras Cineantropom Desempenho Hum 2007;9:339-50.

57. Reis MS, Reis RS, Hallal PC. Validity and reliability of a physical activity social support assessment scale. Rev Saude Publica 2011;45(2):294-301
